# Klotho inhibits neuronal senescence in human brain organoids

**DOI:** 10.1038/s41514-021-00070-x

**Published:** 2021-08-02

**Authors:** Mohammed R. Shaker, Julio Aguado, Harman Kaur Chaggar, Ernst J. Wolvetang

**Affiliations:** Australian Institute for Bioengineering and Nanotechnology, The University of Queensland, St Lucia, QLD Australia

**Keywords:** Neural ageing, Ageing

## Abstract

Aging is a major risk factor for many neurodegenerative diseases. Klotho (KL) is a glycosylated transmembrane protein that is expressed in the choroid plexus and neurons of the brain. KL exerts potent anti-aging effects on multiple cell types in the body but its role in human brain cells remains largely unclear. Here we show that human cortical neurons, derived from human pluripotent stem cells in 2D cultures or in cortical organoids, develop the typical hallmarks of senescent cells when maintained in vitro for prolonged periods of time, and that moderate upregulation or repression of endogenous *KL* expression in cortical organoids inhibits and accelerates senescence, respectively. We further demonstrate that *KL* expression alters the expression of senescence-associated genes including, extracellular matrix genes, and proteoglycans, and can act in a paracrine fashion to inhibit neuronal senescence. In summary, our results establish an important role for KL in the regulation of human neuronal senescence and offer new mechanistic insight into its role in human brain aging.

## Introduction

Klotho (KL) is a type I transmembrane protein with remarkable anti-aging properties^1^, but is progressively downregulated during ageing^2^. The full-length transmembrane form of KL is subject to ectodomain shedding via disintegrin and metalloproteinase (ADAM)− 10 and −17^3^, and beta- and gamma-secretase enzymes^1^, but a secreted form of KL can also be generated via alternative splicing^4^. KL is highly expressed in renal tubules, parathyroid glands, and choroid plexus but is also readily detectable in other tissues, including many brain cell types^4^. In renal proximal tubular epithelium, KL functions as a co-receptor for FGFR1 to regulate FGF23 signaling, and thus regulates phosphate homeostasis and Vitamin D metabolism. KL also performs important hormone-like functions in other tissues, suppressing insulin/IGF1 and Wnt signaling, and promoting increased resistance to oxidative stress^5^ and inflammation^6^. In the brain, KL is predominantly expressed in ependymal cells of the choroid plexus, but is also detected in hippocampal neurons, cortical neurons, cerebral white matter^7^, and cerebellar Purkinje cells^8^. KL expression in murine retinal pigment epithelial cells^9^ or rat hippocampal neurons^10^ enhances cell survival by inhibiting oxidative stress. In agreement with these data, *Klotho*-deficient mice show impaired cognition, while overexpression of *Klotho* in mice increases lifespan by 30–40%^11^, improves cognitive function in young and old mice, and enhances re-myelination in cuprizone-treated mice^12^. In support of the notion that this is at least in part mediated by direct effects on neuronal cells, recombinantly produced KL increases differentiation and maturation of murine oligodendrocytes, and lentiviral delivery of *Klotho* to the brain reduced cognitive deficits in a mouse model of Alzheimer’s disease^13^. In humans, the level of KL in the cerebrospinal fluid was found to decrease with advanced age and in Alzheimer’s disease patients^14^. Conversely, *KL* polymorphisms that lead to a higher KL expression are associated with increased longevity, increased brain volume, and enhanced cognition^4^. Collectively these studies, therefore, suggest that KL may play an important role in preventing brain aging and aging-associated neurodegenerative conditions. However, to date, *KL*’*s* impact and role in human neurons remains unclear. To address this and explore the neural cell autonomous and potential paracrine effects of *KL*, we here utilized human pluripotent stem cell (PSC)-derived neurons and brain organoids. We show that cortical neurons in brain organoids cultured for prolonged periods of time show increased senescence that is accompanied by a reduction in *KL* expression levels. Importantly, we demonstrate that upregulation of endogenous *KL* expression in human PSC-derived brain organoids inhibits multiple hallmarks of senescence whereas transcriptional repression of *KL* enhances senescence. Transcriptome analysis of brain organoids with moderate enforced upregulation of endogenous *KL* expression revealed modulation of senescence, extracellular matrix, and proteoglycan genes. Our data demonstrate that *KL* has direct beneficial effects on human neurons and thus holds significant potential for improving human brain function with advanced age and for treating aging-related neurodegenerative diseases such as Alzheimer’s disease.

## Results

### Increased cellular senescence in human cortical organoids cultured for extended periods of time

**Fig. 1:**
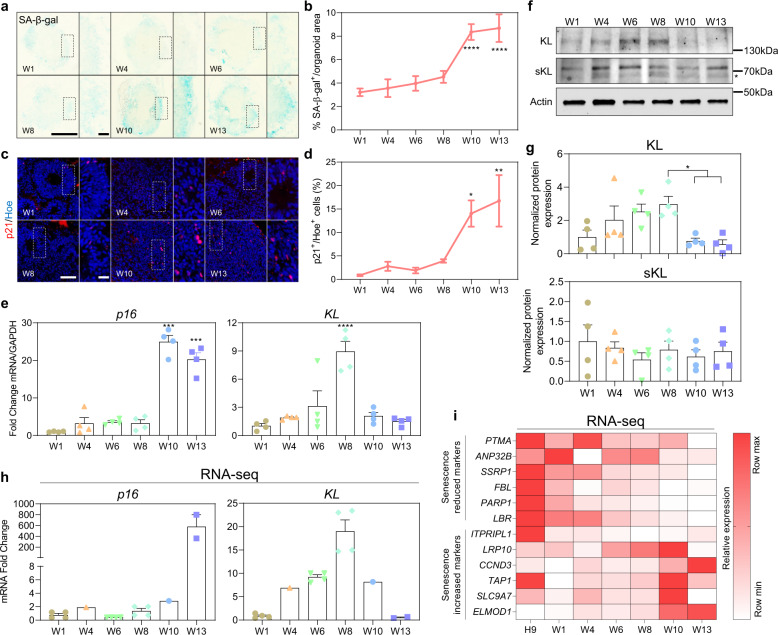
Senescence-related changes in human cortical brain organoids. **a** Representative images of sections of human cortical brain organoids derived from H9-ESCs cultured over 13 weeks in vitro and stained with SA-β-gal. Scale bar = 1000 µm. Dotted box indicates a magnified image. W is week. **b** Quantification of the percentage of SA-β-gal area normalized to the total area of each individual organoid derived from H9-ESCs. Data are presented as mean ± standard deviation. ****P < 0.0001 via one-way ANOVA. Number of independent experiments = 3. Total number of analyzed organoids = 72. W is week. **c** Representative images of sections of different ages of human organoids derived from H9-ESCs. Sections were stained with p21 antibody (red) and counterstained with Hoechst 33342 (blue). Scale bar = 65 µm. W is week. White dotted box indicates the magnified image. **d** Quantification of the percentage of total p21^+^ cells relative to the total number of cells per organoids derived from H9-ESCs. Data are presented as mean ± standard deviation. **P < 0.001, *P < 0.01 via one-way ANOVA. Number of independent experiments = 3. Total number of analyzed organoids = 63. W is week. **e** qRT-PCR of *p16* and *KL* at different stages of in vitro culture of human cortical organoids derived from H9-ESCs. All values were normalized to GAPDH levels of their respective samples, and expressed relative to W1 values to obtain the fold change. Data are shown as mean ± standard error mean; Number of independent experiments = 4. Total number of analyzed organoids = 72; ***P < 0.001, ****P *<* 0.0001 via one-way ANOVA. W is week. **f** Western blots showing the protein levels of full-length KL and secreted KL in human cortical organoids derived from H9-ESCs cultured over 13 weeks. Actin was used for normalization. Star indicates the specific band of secreted KL, sKL indicates secreted KL. All blots derive from the same experiment and processed in parallel. **g** Bar graphs show the quantification of KL, as well as secreted KL levels obtained from **f**. Data are shown as mean ± standard error mean. The number of independent experiments = 4, total number of examined organoids is 72. sKL indicates secreted KL. **h** Normalized relative expression values of *p16 and KL* mRNA obtained from published RNA-seq studies. The fold change was calculated by dividing the normalized mRNA value of different weeks over W1. Data are shown as mean ± standard error mean. W is week. **i** Heatmap represents the expression of RNA-seq data of senescence increased and decreased markers in cortical organoids derived from iPSCs cultured over different time points. Relative expression data were further normalized between 0 (lowest) to 1 (highest) expression value of individual genes across different time points of organoids culture. Normalized expression values were color-coded with red values indicating upregulation and white values indicating downregulation. W is week.

Human pluripotent stem cell-derived brain organoids largely recapitulate the developmental trajectories, cellular make-up, and architecture of the developing human brain^15^. Since in vitro culture of organoids imposes significant cellular and metabolic stress^16^, we hypothesized that prolonged culture would promote senescence of neural cell types within human brain organoids. To test this, we generated cortical organoids from H9 embryonic stem (ES) cells and cultured these for 1, 4, 6, 8, 10, and 13 weeks (Supplementary Fig. 1a). Quantification of the number of cells that exhibit senescence-associated β-galactosidase (SA-β-gal) activity in these cortical organoids revealed a gradual increase in SA-β-gal positive cells over time in culture with a large and significant induction at weeks 10 and 13, as compared to earlier time points (Fig. 1a, b). This was concomitant with a significant increase in the percentage of cells that express p21^CIP1/WAF1^ (CDKN1A) protein (Fig. 1c, d and Supplementary Fig. 1b), and with increased expression of *p16*^*INK4A*^ mRNA (Fig. 1e) at weeks 10 and 13, both markers associated with cellular senescence^17^. Interestingly, mRNA expression of *KL* steadily increased between weeks 1 and 8 of organoid culture, but was then dramatically downregulated at weeks 10 and 13 (Fig. 1e), when the onset of senescence was most clearly observed in the cortical brain organoids (Fig. 1a–d). In agreement with these data, the protein level of full-length KL was gradually and significantly increased up to week 8, but next sharply and significantly dropped at weeks 10 and 13 (Fig. 1f, g). The protein expression of the secreted form of KL (sKL) did not significantly change over the cortical brain organoid differentiation time course (Fig. 1f, g). Collectively, these data led us to conclude that prolonged culture of human pluripotent ES cell-derived cortical brain organoids results in protein and gene expression changes suggestive of senescence that coincide with downregulation of full-length KL.

To independently validate these data, we next examined published RNA-seq datasets GSE82022^18^, GSE110006^19^, and GSE97881^20^ derived from cortical brain organoids also cultured for at least 13 weeks. In agreement with our observations, these datasets also showed increased expression of *KL* mRNA in cortical organoids cultured for 1, 4, 6, and 8 weeks (Fig. 1h), that was followed by a sharp decrease by weeks 10 and 13, as senescence genes such as *p16*^*INK4A*^ were upregulated (Fig. 1h). We then filtered these databases for genes that are consistently up and downregulated in senescence^21^. This revealed a progressive downregulation of mRNA expression of genes typically downregulated in senescence such as *PTMA*, *ANP32B*, *SSRP1*, *FBL*, *PARP1*, *LBR*, and *ITPTIPL1*^21^, (Fig. 1i), and an increase in senescence-associated genes such as *LRP10*, *CCND3*, *TAP1*, *SLC9A7*, and *ELMOD1*^21^ in 10–13-weeks-old cortical brain organoids (Fig. 1i). Collectively, we interpreted these data to indicate that human stem cell-derived cortical organoids cultured for at least 10 weeks show downregulation of KL, contain increasing amounts of cells that exhibit the typical hallmarks of senescence, and therefore constitute a useful model to study the impact of KL on senescence of human neural cell types in vitro.

### Upregulation of endogenous *KL* inhibits culture-induced senescence in human cortical brain organoids

**Fig. 2:**
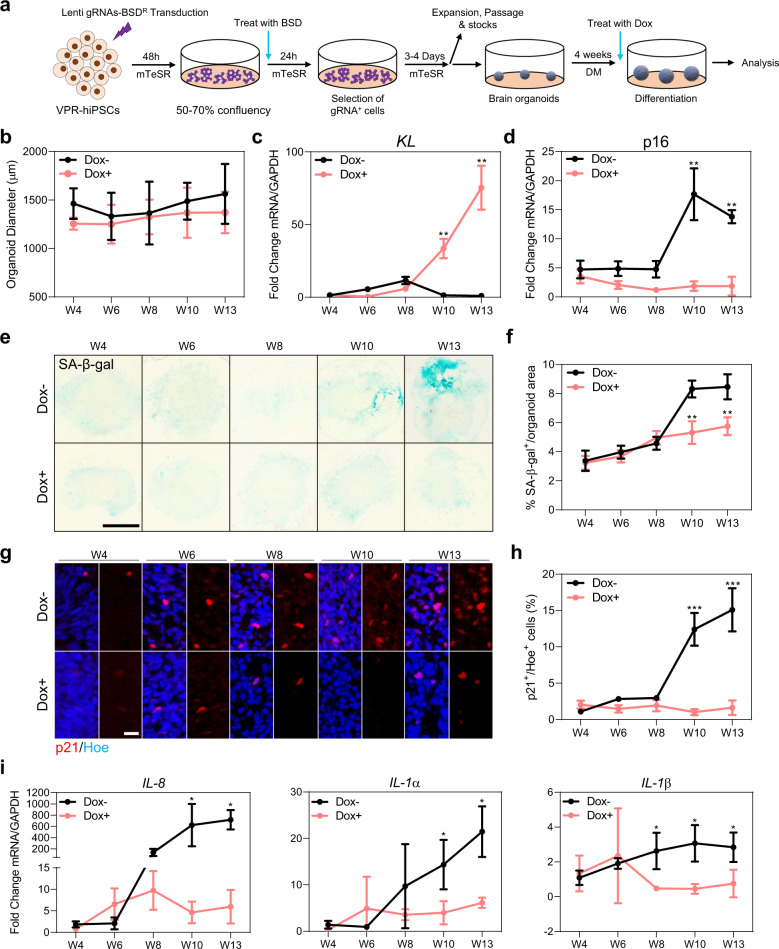
*KL* overexpression modulates senescence-related changes in human cortical brain organoids. **a** Schematic diagram showing the experimental plan used to endogenously activate *KL* using WTC iPSCs-VPR cell line upon daily dox treatment at weeks 4–13. W is week, *KL* is Klotho. **b** Measurement of the diameter of organoids derived from human dCas9-VPR iPSCs with and without dox treatment from weeks 4 to 13. Data are the mean ± standard deviation. Number of independent experiments *N* = 4. Total number of analyzed organoids = 78. **c** qRT-PCR of *KL* at different stages of in vitro culture of human cortical brain organoids derived from human dCas9-VPR iPSCs following daily treatment with dox from weeks 4 to 13. Dox− represents organoids without dox treatment. All values were normalized to GAPDH levels of their respective samples, normalized data were further normalized to W4 to obtain the fold change value. Data are shown as mean ± standard error mean; Number of independent experiments *N* = 4. Total number of analyzed organoids = 96; **P < 0.001 via One-Way ANOVA. W is week. **d** qRT-PCR of p16 at different stages of in vitro culture of human cortical organoids derived from human dCas9-VPR iPSCs following daily dox treatment from weeks 4 to 13. KL− represents organoids without dox treatment. All values were normalized to GAPDH levels of their respective samples, normalized data were further normalized to W4 to obtain the fold change value. Data are shown as mean ± standard error mean; Number of independent experiments = 4. Total number of analyzed organoids = 96; **P < 0.001 via one-way ANOVA. W is week. **e** Representative images of SA-β-gal stained sections of human cortical brain organoids derived from dCas9-VPR iPSCs transduced with 3 gRNAs and treated with dox+ from weeks 4 to 13. Dox− represents organoids without dox treatment. Scale bar = 1000 µm. W is week. **f** Quantification of the percentage of SA-β-gal area normalized to the total area of each individual organoid derived from human iPSCs. Data are presented as mean ± standard deviation. **P < 0.001 via one-way ANOVA. Number of independent experiments *N* = 4. Total number of analyzed organoids = 80. W is week. **g** Representative images of sections of human cortical brain organoids of different ages derived from human dCas9-VPR iPSCs transduced with 3 gRNAs and treated with dox from weeks 4 to 13. Dox− represents organoids without dox treatment. Tissues were stained with p21 antibody (red). All sections were counterstained with Hoechst 33342 (blue). Scale bar = 100 µm. W is week. **h** Quantification of the percentage of total p21^+^ cells relative to the total number of cells per organoids derived from human dCas9-VPR iPSCs. Data are presented as mean ± standard error mean. ***P < 0.0001 via One-Way ANOVA. Number of independent experiments *N* = 4. Total number of analyzed organoids = 80. W is week. **i** qRT-PCR of SASP at different stages of human cortical organoid derived from human dCas9-VPR iPSCs in vitro culture following daily dox treatment from weeks 4 to 13. Dox− represents organoids without dox treatment. All values were normalized to GAPDH levels of their respective samples, normalized data were further normalized to W4 to obtain the fold change value. Data are shown as mean ± standard error mean; *P < 0.05 and via *t*-test. Number of independent experiments = 4, total number of examined organoids is 144.

Although the anti-aging properties of KL are well documented, its impact on human brain cells remains largely unclear. Having established that human embryonic stem cells-derived cortical brain organoids display a marked increase in senescence between weeks 10 and 13 that coincides with downregulation of KL, we next examined whether enforced expression of *KL* would affect the upregulation of senescence markers such as SA-β-gal, *p16*, and p21 in cortical organoids cultured for extended periods of time. To achieve this, we took advantage of human iPSCs that were engineered to possess a doxycycline (dox)-inducible dCas9-VPR cassette targeted to the AAVS1 safe harbor site. The VPR transcriptional activator can be directed to a promoter of choice with gRNAs and thus enables dox-dependent upregulation of a gene of interest, while maintaining cell-type appropriate splicing. We identified three gRNAs designed to target sites 12 base pairs (bp), 25 and 87 bp upstream of the *KL* promoter start site (Supplementary Fig. 2a) that, when combined, elicited a 1.5-fold increase in KL protein expression in dCas9-VPR iPSCs following seven days of culture with 1 μg/ml dox (Supplementary Fig. 2b, c). We next established cortical brain organoids from dCas9-VPR WTC iPSCs lentivirally transduced with these gRNAs and, in order to prevent potential impacts of *KL* overexpression on the differentiation or patterning of human cortical brain organoids, we activated *KL* expression with dox from week 4 onwards (Fig. 2a). We found that induction of *KL* from week 4 onwards did not significantly affect the size (Fig. 2b) or cellular make-up (Supplementary Figs. 5 and6) of cortical brain organoids following extended time in culture. Enforced *KL* expression (Fig. 2c), however, significantly reduced the levels of *p16*^*INK4A*^ expression in cortical organoids cultured for 10 and 13 weeks, as compared to organoids without dox (untreated) (Fig. 2d). Furthermore, endogenous activation of *KL* reduced the accumulation of SA-β-gal expressing cells observed in 10- and 13-weeks cultured cortical brain organoids compared to the untreated group (Fig. 2e, f). A similar reduction in SA-β-gal expressing cells was also achieved using in house generated dCas9-VPR EU79 iPSCs (Supplementary Fig. 3a), indicating this *KL*-mediated inhibition of senescence is not altered in a different genetic background. Upregulation of *KL* in cortical organoids cultured for 10- and 13-weeks also significantly reduced the number of cells expressing p21 (Fig. 2g, h and Supplementary Fig. 3b, c). In agreement with these data, we found that *KL* upregulation significantly inhibited the mRNA expression levels of a number of Senescence-Associated Secretory Phenotype (SASP) genes (namely *IL-8*, *IL-1α*, and *IL-1β*), whereas these genes were significantly upregulated between weeks 8 and 13 in cortical brain organoids cultured in the absence of dox (Fig. 2i, and Supplementary Fig. 2d). We concluded that moderate upregulation of endogenous expression of *KL* is sufficient to prevent senescence-related changes in human cortical brain organoids cells cultured for extended periods of time in vitro.

### KL inhibits senescence of cortical plate neurons in human cortical brain organoids

**Fig. 3:**
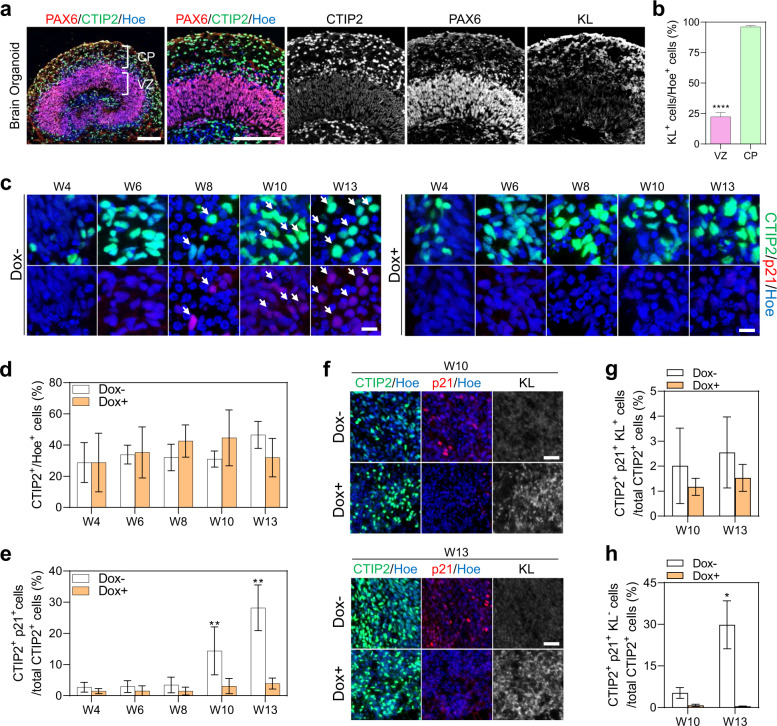
KL expression in the cortical plate and rescue of cortical neuronal senescence. **a** Representative images of sections of 8-week-old cortical organoids derived from human dCas9-VPR iPSCs, immunostained for ventricle zone PAX6 (red) and cortical plate CTIP2 (green) and KL (gray). All sections were counterstained with Hoechst 33342 (blue). Scale bar = 150 µm. VZ is ventricle zone, CP is cortical plate. **b** Percentage of KL^+^ cells relative to the total number of ventricular zone or cortical plate cells per organoid. Data are presented as mean ± standard deviation. ****P < 0.0001 via Student’s *t*-test. Number of independent experiments = 3. Total number of examined organoids = 9. VZ is ventricular zone, CP is cortical plate. **c** Representative images of 4 to 13-week-old cortical brain organoid sections immunostained for cortical neurons CTIP2 (green) and p21 (red). All sections were counterstained with Hoechst 33342 (blue). Dox− indicates cortical brain organoids without dox treatment. Dox+ indicates cortical organoids with daily dox treatment. Scale bar =  15 µm. White arrows indicate the cells expressing CTIP2 and p21. **d** Percentage of CTIP2 + neurons relative to total cells per organoid in organoids with (Dox+) and without KL induction (Dox−). Data are presented as mean ± standard error mean. Number of independent experiments *N* = 4. Total number of analyzed organoids = 80. W is week. **e** Percentage of CTIP2 + neurons expressing p21 relative to total cells per organoid in organoids with (Dox+) and without KL induction (Dox−). Data are presented as mean ± standard error mean. **P < 0.001 via one-way ANOVA. Number of independent experiments *N* = 4. Total number of analyzed organoids = 80. W is week. **f** Representative images of sections of weeks 10 and 13-old cortical brain organoid derived from human dCas9-VPR iPSCs, immunostained for cortical neurons CTIP2 (green), p21 (red), and KL (gray). All sections were counterstained with Hoechst 33342 (blue). Dox− indicates cortical brain organoids without dox treatment. Dox+ indicates cortical organoids with daily dox treatment. Scale bar = 20 µm. **g** Percentage of CTIP2 + p21+ KL + neurons relative to total CTIP2 + neurons per organoid in organoids with (Dox+) and without KL induction (Dox−). Data are presented as mean ± standard deviation. Number of independent experiments *N* = 3. Total number of analyzed organoids = 24. W is week. **h** Percentage of CTIP2 + p21+ KL− neurons relative to total CTIP2 + neurons per organoid in organoids with (Dox+) and without KL induction (Dox−). Data are presented as mean ± standard deviation. *P < 0.05 via Student’s *t*-test. Number of independent experiments *N* = 3. Total number of analyzed organoids = 24. W is week.

We next sought to understand the cell type-specific roles of *KL* in human cortical organoids. At 8 weeks in vitro, cells within cortical brain organoids segregate into ventricular zone (VZ) progenitors that express PAX6, and cortical plate (CP) zone neurons that express CTIP2^22^ (Fig. 3a). We found that KL protein expression was readily detected in 96% of CP cells, whereas only 22% of VZ cells expressed low amounts of KL protein (Fig. 3a, b), and in these cells was predominantly confined to progenitors positioned at the apical domain (Fig. 3a).

To examine whether KL expression in cortical neurons impacts their entry into senescence, we generated cortical brain organoids and endogenously overexpressed *KL* as illustrated in Fig. 2a. We double-labeled sections of dox-treated and untreated cortical brain organoids with CTIP2 and p21 antibodies (Fig. 3c). Automated quantification of staining revealed that enforced expression of endogenous *KL* did not significantly alter the proportion of CTIP2^+^ neurons in cortical brain organoids (Fig. 3d), but significantly reduced the upregulation of p21 observed in CTIP2^+^ neurons in cortical brain organoids cultured in the absence of dox (Fig. 3e). To explore to what extent this *KL*-mediated inhibition of senescence of CTIP2^+^ neurons was due to cell autonomous or paracrine effects, we examined weeks 10 and 13 time points, when p21 expression is significantly induced, and quantified the percentage of CTIP2^+^ p21^+^ KL^+^ vs CTIP2^+^ p21^+^ KL^−^ cells in cortical brain organoids with and without dox treatment (Fig. 3f–h). While we found a low and insignificant difference in the amount of CTIP2^+^ p21^+^ KL^+^ cells between dox-treated and untreated organoids (Fig. 3g), there was a significant reduction in CTIP2^+^ p21^+^ KL^−^ in dox-treated cortical brain organoids compared to the untreated group (Fig. 3h). It should be noted that not all CTIP2^+^ neurons are KL^+^ in these organoids (Supplementary Fig. 4a). Collectively, these data first indicate that moderate upregulation of *KL* expression in CTIP2^+^ neurons inhibits their entry into senescence, and that activation of *KL* in cortical brain organoids significantly protects CTIP2^+^ cortical neurons that are KL negative from becoming senescent, suggesting KL acts through paracrine signaling to bystander cells. To further substantiate the causal relationship between KL and cortical neuronal senescence, we next utilized the Gen2C iPSCs-KRAB cell line that was engineered to direct the KRAB transcriptional repressor to a promoter of choice with gRNAs upon dox treatment. One gRNA was sufficient to endogenously suppress *KL* expression upon treatment with dox over 7 days (Supplementary Fig. 4b). Repression of *KL* in brain organoids from week 4 onwards moderately but significantly reduced brain organoid size over time in culture (Supplementary Fig. 4c, d), in agreement with the observation that *kl*/*kl null* mice display a severe growth deficit, including the brain^23^. Furthermore, enforced transcriptional downregulation of *KL* resulted in a significant upregulation of *p16*^*INK4A*^ (Supplementary Fig. 4e), and an increase in senescent cortical neurons in 8–13 weeks of cultured organoids (Supplementary Fig. 4f), indicating that KL is necessary to prevent premature neuronal senescence.

Cortical brain organoids contain neural progenitors in addition to neurons, hence, we next examined whether neural progenitors exhibited senescence phenotypes in these organoids. We found 5% of all SOX2^+^ neural progenitors co-stained with p21 protein between W8 and W13 (Supplementary Fig. 5a, b), and that this was not inhibited by *KL* overexpression. We also failed to detect changes in the proportion of SOX2^+^ cells population in the absence or presence of enforced *KL* expression (Supplementary Fig. 5c, d). After neurogenesis has been initiated, cortical brain organoids are known to exhibit waves of gliogenesis^24^, and we similarly observed the specification of GFAP expressing cells in our organoids (Supplementary Fig. 6a). We found that dox-induced upregulation of *KL* did not affect the proportion of GFAP^+^ cells in the organoids (Supplementary Fig. 6b, c). Collectively, these data indicate that in pluripotent stem cell-derived cortical brain organoids KL is predominately expressed in cortical neurons and that these neurons are more susceptible to senescence than other neural cells when cultured for extended periods of time. Our data further show that moderate upregulation of endogenous *KL* expression in human cortical brain organoids is necessary and sufficient to prevent human cortical neuronal cell senescence via paracrine mechanisms but has little effect on neural progenitor cells in this in vitro model.

**Fig. 4:**
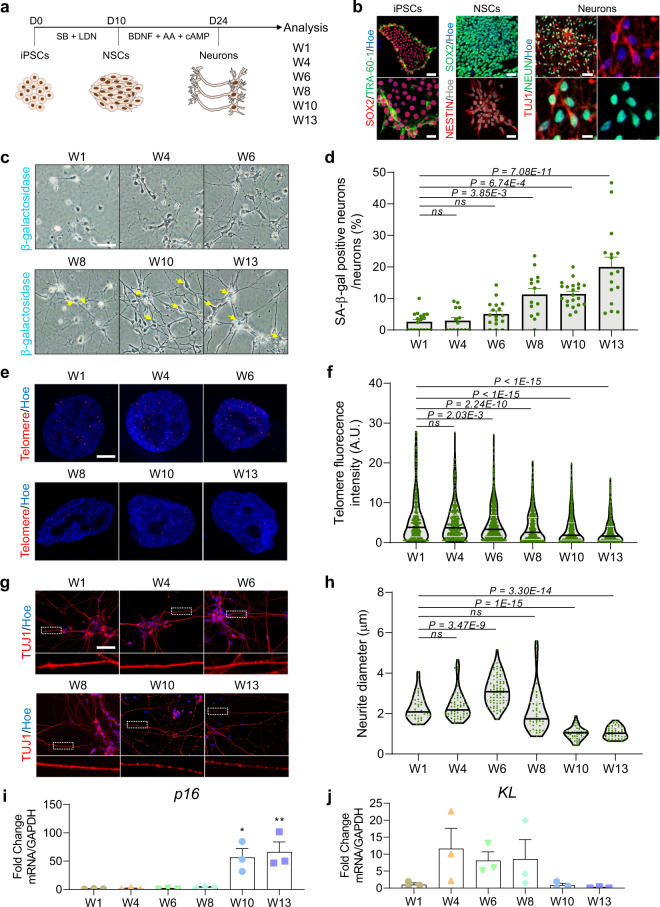
Human H9-ESCs derived neurons exhibit hallmarks of senescence in vitro. **a** Schematic representation of the strategy used to generate cortical neurons via neural stem cells (NSCs) derived from H9-ESCs. **b** Representative images of H9-ESCs immunostained with SOX2 (red), TRA-60 (green). Neural stem cells (NSCs) immunostained with SOX2 (green), NESTIN (red). Double labeling of the iPSC-derived human neurons with NEUN (green) and TUJ1 (red). All cells were counterstained with Hoechst 33342 (blue/gray). Scale bar = 50 µm. Zoomed image scale bar = 10 µm. **c** Representative images of SA-β-gal staining of human H9-ESCs derived neurons in vitro cultured for up to 13 weeks. Scale bar = 100 µm. W is week. **d** Quantification of the percentage of SA-β-gal^+^ neurons normalized to the total number of neurons per field of view. Data are presented as mean ± standard deviation. *P*-value is indicated in the graph via One-Way ANOVA. Number of independent experiments = 3. W is week. **e** Representative image of telomere staining of neuronal nuclei using fluorescence in situ hybridization (FISH). All cells were counterstained with Hoechst 33342 (blue). W is week. Scale bar = 2.5 µm. **f** Quantification of telomere staining intensity. Data are presented as mean ± standard deviation. *P*-value is indicated in the graph via one-way ANOVA. Number of independent experiments = 3. W is week. **g** Representative images of human H9-ESCs derived neurons cultured for up to 13 weeks. Neurons were stained with TUJ1 antibody (red). All cells were counterstained with Hoechst 33342 (blue). Zoomed images below show the morphology and thickness of the axons. Scale bar = 25 µm. W is week. **h** Quantification of neurite diameter of human iPSC-derived neurons cultured for up to 13 weeks. Data are presented as mean ± standard error mean; *P*-value is indicated in the graph via One-Way ANOVA. Number of independent experiments = 3, W is week. **i** qRT-PCR of p16 at different stages of in vitro human neuronal differentiation. All values were normalized to GAPDH levels of their respective samples, and expressed relative to W4 values to obtain the fold change. Data are shown as mean ± standard error mean; Number of independent experiments = 3; *P < 0.05, **P < 0.01 via one-way ANOVA. W is week. **j** qRT-PCR of *KL* at different stages of in vitro human neuronal differentiation. All values were normalized to GAPDH levels of their respective samples, and expressed relative to W4 values to obtain the fold change. Data are shown as mean ± standard error mean; Number of independent experiments = 3. W is week.

Having established that cortical neurons in brain organoids display typical features of senescence after the prolonged culture that can be modulated by *KL* expression, we next investigated this process in 2D neuronal cultures. To this end, we generated 2D neurons derived from iPSC-neural stem cells (NSCs) and cultured these up to 13 weeks before analysis (Fig. 4a, b). Remarkably, similar to what we observed in cortical brain organoids, iPSC-derived neurons again exhibited a progressive increase in the proportion of neurons that show SA-β-gal activity from week 6 onwards which increased to ~20% of neurons by week 13 (Fig. 4c, d). Because telomere shortening is an established hallmark of senescent cells^25^, even in post-mitotic cells such as neurons^26^, we next measured telomere length by fluorescence in situ hybridization (FISH) (Fig. 4e). This revealed that human iPSC-derived neurons exhibited a progressive reduction in telomere length between 6 and 13 weeks of in vitro culture (Fig. 4f). Since the thickness of neuronal axons indicates the vulnerability of neurons to toxicity, we next quantified the neurite diameter of neurons over time in culture, revealing a significant loss of axon thickness between weeks 8 and 13 (Fig. 4g, h). Similar to cortical brain organoids, human neurons also exhibited increased expression of *p16* ^*INK4A*^ mRNA (Fig. 4i) at weeks 10 and 13. This was concomitant with a reduction in *KL* mRNA expression at weeks 10 and 13 (Fig. 4j), when the onset of senescence was most clearly observed in 2D human neurons. Collectively, these data indicate that extended in vitro culture-induced senescence-like phenotypes in 2D human neurons and that this is also accompanied by *KL* downregulation.

### Differential expression of anti-senescence genes in cortical brain organoids upon *KL* overexpression

**Fig. 5:**
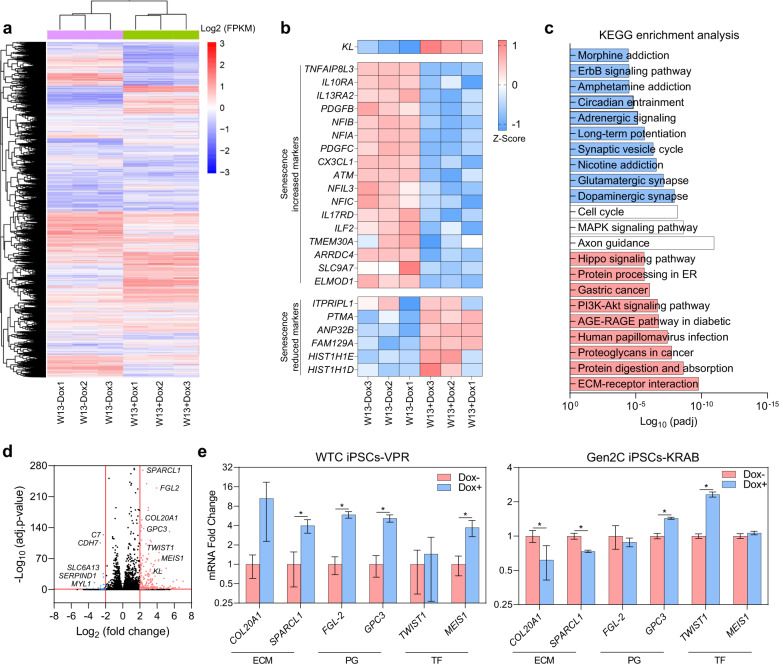
Transcriptome analysis of cortical brain organoids with *KL* overexpression identifies enrichment for ECM and proteoglycan genes. **a** Heatmap expression patterns of 13-weeks cortical brain organoid with *KL* overexpression compared to control/untreated organoids. **b** Heatmap representing the expression of senescence-associated markers as well as senescence increased and decreased markers in cortical brain organoids derived from human dCas9-VPR iPSCs cultured over 13 weeks with/without *KL* overexpression. Normalized expression values were color-coded with red values indicating upregulation and white values indicating downregulation. W is week. **c** Bar graphs showing KEGG enrichment analysis of upregulated genes (red), downregulated genes (blue), and unaffected genes (white) in 13-weeks cortical brain organoids compared with untreated organoids. **d** Volcano plot highlighting differentially expressed genes in 13-weeks cortical brain organoid with *KL* overexpression. Red color defines an upregulated expression with a log_2_ (fold change) > 2, blue defines a downregulated expression with a log_2_ (fold change) < 2. **e** Fold change of mRNA levels of genes identified in **d**. Left panel represents data derived from WTC iPSCs-VPR, right panel represents data derived from Gen2C iPSCs-KRAB. All values were normalized to GAPDH levels of their respective samples and expressed relative to Dox− 13-weeks cortical brain organoids values to obtain the fold change. Data are shown as mean ± standard deviation. Number of independent experiments = 3, total number of analyzed organoids = 72; *P < 0.05 via Student’s *t*-test. W is week.

To obtain insight into the mechanisms via which KL protects cortical neurons from entry into senescence, we compared the transcriptomes of week 13 cortical organoids with and without *KL* overexpression (Fig. 5a). We found 2663 genes that were significantly upregulated (Supplementary Tables 1) and 2608 downregulated genes (Supplementary Tables 2) upon overexpression of *KL* in cortical brain organoids. Hierarchical clustering of differentially expressed genes revealed several co-regulated genes cohorts and confirmed the suppression of senescence-associated genes upon *KL* overexpression in cortical brain organoids (Fig. 5b), previously identified by qPCR and IHC analysis^21^. KEGG enrichment analysis revealed enrichment of many extracellular matrix (ECM) and proteoglycans-related genes upon *KL* overexpression in cortical brain organoids (Fig. 5c), with 33.5% of ECM genes among the top 10% of upregulated genes (Supplementary Fig. 6d). Control cortical brain organoids were enriched for synapse and addiction-associated genes including nicotine, amphetamine, and morphine (Fig. 5c), many of which are known to be upregulated in senescence (Supplementary Table 3)^27^. Genes with no significant changes between the two groups were associated with axon guidance and cell cycle (Fig. 5c), suggesting that modulation of *KL* expression has no significant impact on differentiation and proliferation processes. In addition, we found that *KL* has no effect on the cellular composition of cortical organoids as indicated by equivalent expression levels of neural cell markers between organoids with and without dox (Supplementary Fig. 6e). Filtering for genes with a cut-off value of >2-fold change and an adjusted *P*-value < 0.05 identified 233 upregulated genes (Volcano plot shown in Fig. 5d, gene list in Supplementary Table 4). Interaction analysis of this gene list with *KL* using GeneMANIA identified 6 genes including two ECM genes (*SPARCL1* and *COL20A1*)^28^, two transcription factors (*TWIST1*^29^ and *MEIS1*^30^), and two proteoglycan genes (*FGL2*^31^ and *GPC3*^32^), all of which are known to be involved in inhibition of senescence^28–32^. Significantly strong increased expression of *SPARCL1*, *FGL-2*, *GPC3*, and *MEIS1* genes in week 13 cortical organoids with *KL* overexpression was next validated by qRT-PCR (Fig. 5e). Interestingly, the dox-induced suppression of *KL* expression in cortical organoids derived from KRAB-iPSCs significantly inhibited the expression of *COL20A1* and *SPARCL1* only (Fig. 5e). Collectively, these results revealed the enrichment of ECM in cortical brain organoids upon *KL* overexpression, and identified several anti-senescence genes that are co-regulated with *KL*, further supporting the notion that *KL* contributes to inhibition of senescence in human cortical neurons via modulation of ECM make-up, in agreement with its paracrine mode of action identified in this study.

## Discussion

Cortical brain organoids have become an invaluable model platform for human brain disease modeling^33^, as they reproducibly generate the complex neuronal networks and cell diversity of the developing human cerebral cortex and can recapitulate aging-related neurodegenerative processes such as β-amyloid aggregation^34^, and Tau-pathology of Alzheimer’s disease^35^ in vitro. Gene inactivation and overexpression studies in mice previously showed that *KL* increases lifespan^11^, enhances myelination, synaptic plasticity, and cognitive functions, and protects neuronal cultures against Aβ and excitotoxicity^10^. Its effect on human brain cells has however remained under-investigated, despite the fact that heterozygous carriers of the “KL-VS” KL gene variant, who exhibit higher levels of circulating sKL, display extended longevity^36^, larger brain volumes^37^, and enhanced cognition^37^. Because the extracellular domain of sKL can be proteolytically released and then act as a hormone on remote tissues, the direct impact of KL expression in the human central nervous system has been particularly difficult to investigate. To address this, we here generated human neurons and brain organoids from human iPSC and H9-ESC. We first demonstrate that both cortical neurons differentiated from human iPSC in 2D cell culture and in 3D cortical brain organoids show the typical hallmarks of senescent cells such as increased SA-β-gal activity, increased expression of p16 and p21, increased secretion of inflammatory cytokines, and accelerated telomere shortening, provided they are cultured for extended periods of time. Importantly, we demonstrate that the emergence of these hallmarks of senescence coincides with a sharp downregulation of *KL* between weeks 10–13 of culture. Previously, it was shown that accelerated senescence can be detected in iPSC-derived cells such as smooth muscle cells derived from Hutchinson–Gilford progeria syndrome iPSCs^38^, and in neurons from human iPSC that artificially overexpress Progerin^39^. Our data now show that despite the well-established epigenetic rejuvenation that accompanies reprogramming to pluripotency^40^, even healthy control pluripotent stem cell-derived neurons can become senescent and display typical hallmarks of aging provided they are cultured for sufficiently long periods of time. Our data are in agreement with previous reports that demonstrate that increased organoid size limits the diffusion and transport of nutrients, growth factors, and oxygen and that this negatively impacts cell type specification, progenitor maturation, and cell viability^9,15,16,41^. Our data may indeed provide a potential explanation as to why disease processes that in vivo emerge much later in life, such as β-amyloid aggregation and tau-pathology, can be readily detected in transcriptionally and epigenetically “young” brain organoids^34,35^.

We show that KL is expressed in CTIP2^+^ cortical neurons in human brain organoids. This is in agreement with a previous study showing *KL* mRNA and protein distribution in rat brain by in situ hybridization and immunohistochemistry, respectively^42^, and with the observation that in human post-mortem brain KL is primarily detected in neuronal cells of the cerebral cortex, Purkinje cells, and in motor neurons in the gray matter of the ventral horns of the spinal cord^8^. sKL is generated upon cleavage of full-length KL that is dependent on ADAM activity^43^. While we detected a significant reduction in full-length KL at W10 and W13 during the neuronal senescence induction period in cortical brain organoids, we found no changes in endogenous sKL production overtime, suggesting a minimal contribution of sKL in cortical neuronal senescence of these organoids. To investigate the functional role of KL in human neuronal cell types, we used human pluripotent stem cells with an AAVS-targeted dox-inducible dCas9-VPR transcriptional activator cassette and lentivirally delivered gRNAs targeting the upstream of *KL* promoter, enabling us to upregulate endogenous expression of *KL* in neural cells at will. Our data show that moderate 2-fold upregulation of *KL* effectively inhibits increased expression of the senescence markers p21 and p16 and elevated SA-β-gal activity in human cortical neurons. Because our *KL* PCR primers detect all three forms of α- *KL*, it remains to be determined whether it is the transmembrane, processed, or spliced forms of *KL* that are essential for suppressing senescence in neuronal cells. Irrespectively, our data strongly suggest that KL’s protective and senescence inhibitory effects in cortical neurons are mediated by cell autonomous as well as paracrine signaling and likely ECM modulating effects of *KL*. While we recognize that in vivo KL may also act via hormonal or metabolic effects brought about by secreted KL from other peripheral organs such as the kidney^44^, our data show unequivocal direct effects of KL on brain cell types and in particular on cortical neurons. One of the limitations of current organoids culture systems is the limiting penetration of oxygen and nutrition to the core of the organoids as they become bigger over time, which will eventually result in hypoxia and necrosis^41^. Both hypoxia and necrosis are known to induce cellular senescence^45^, and we considered the possibility that this may underlie the increase in senescence observed in weeks 10–13 organoids. However, despite the fact that our organoids indeed possess a hypoxic and necrotic core at week 13, we predominantly observe SA-β-gal activity and p21 expression in the non-hypoxic periphery of the organoids, indicating that the hypoxic core is unlikely to underline the increased senescence that accompanies *KL* downregulation at these time points.

Transcriptome analysis of cortical organoids at week 13 with and without *KL* overexpression revealed that expression of ECM molecules may be an important contributor to the anti-aging function of KL in cortical neurons. This is consistent with the notion that ECM make-up is recognized as an important contributor to the senescent phenotype^46^. Differential expression gene analysis further narrowed down the list of upregulated genes (*SPARCL1, FGL2, COL20A1, GPC3, TWIST1*, and *MEIS1*) upon *KL* overexpression. Interestingly, only *SPARCL1* and *COL20A1* were downregulated upon *KL* repression in organoids derived from the Gen2C iPSCs-KRAB line, where both *SPARCL1* and *COL20A1* are ECM genes that are potentially associated with senescence^28^. This transcriptomic analysis provides further mechanistic insights into putative signaling pathways controlled by *KL*, which might be involved in KL-mediated functions in cortical neurons. Furthermore, our data provide support for the idea that molecular targeting of *KL* at the translational level may represent an effective treatment strategy for preventing neuronal senescence in humans. The in vitro model system reported here provides a tractable experimental platform for identifying molecules that can achieve this and for dissecting the molecular processes that mediate *KL*’s beneficial effects on the human brain.

## Methods

### Human embryonic stem cells culture and cortical organoids generation

Human embryonic stem cells H9 from (Wisconsin International Stem Cell Bank, WiCell Research Institute, WA09 cells), WTC iPSC (gift from Professor Bruce Conklin), EU79 iPSCs^47^, and KRAB-GEN2C iPSCs lines^48^ were cultured according to Stem Cell Technologies protocols on feeder-free in hESCs medium on Matrigel (StemCell Technologies, Cat. #354277) in mTeSR (Stem Cell Technologies, Cat. #85851)^49^. The generation of cortical organoids was essentially performed as described previously^33,50^ with modifications. In brief, patterned embryoid bodies were further expanded for four days in N2 medium: DMEM/F12 (Gibco, Cat. #11320-33), 2% B-27 supplement (Gibco, Cat. #17504044), 1% N-2 supplement (Gibco, Cat. #17502-048), 1% MEM Non-Essential Amino Acids (Gibco, Cat. #11140-050), 1% penicillin/streptomycin (Gibco, Cat. #15140148), 0.1% β-mercaptoethanol (Gibco, Cat. #21985-023), embryoid bodies were supplemented daily with (bFGF, 20 ng/ml; R&D, Cat. #233-FB-01M). Embryoid bodies with a clear neuroepithelial edge were selected for Matrigel embedding. Embryoid bodies were then embedded in Matrigel (StemCell Technologies, Cat. #354277) and switched in the terminal differentiation medium DMEM-F12 (Gibco, Cat. #11320-33): Neurobasal medium (Gibco, Cat. #A35829-01) 1% N2 (Gibco, Cat. #17502-048), 12.5 µl of insulin (Sigma), 2% GlutaMAX, 1% MEM Non-Essential Amino Acids (Gibco, Cat. #11140-050), 1% penicillin/streptomycin (Gibco, Cat. #15140148), 0.1% β-mercaptoethanol (Gibco, Cat. #21985-023), and 2% B-27 supplement (Gibco, Cat. #17504044). Fresh media was replaced three times a week. All experiments were carried out in accordance with the ethical guidelines of the University of Queensland and with the approval by the University of Queensland Human Research Ethics Committee (Approval number-2019000159).

### Generation of stably transduced VPR and KRAB lines

Three gRNAs were designed to endogenously overexpress *KL* (gRNA1 Forward sequence: GCATAAAGGGGCGCGGCGCG, gRNA2 Forward sequence: CGGCGGGGCGCGGGCATAAA, gRNA3 Forward sequence: TTATTGCCACGGAGCCCGC), and two gRNAs were designed to endogenously repress *KL* expression (gRNA1 Forward sequence: GAGCGCCGCCTACCAGACCGA, gRNA2 Forward sequence: GCGTTCCGGGAGTCTCCCGG). The annealed gRNAs were then cloned into the Plenti V5 Topo BSD selection cassette vector driven by the U6 promoter. The production of lentivirus was achieved by co-transfection of the cloned Plenti V5 Topo BSD selection cassette vector along with the VSV-G (Addgene, Plasmid #14888) and psPAX2 (Addgene, Plasmid #12260) into 293FT cells (ThermoFisher Scientific, Cat. #R70007) using the Lipofectamine 3000 (Thermofisher Scientific, Cat. #L3000008). Lentivirus particles were then transduced into the dCas9-VPR iPSCs, and appropriate antibiotic selection was carried on three days after transduction. During the culture, transduced dCas9-VPR iPSCs were occasionally exposed to appropriate antibiotic selection to maintain a high proportion of dCas9-VPR iPSCs having *KL* gRNA.

### Generation of human neurons

Human iPSCs were cultured in feeder-free maintenance media (mTeSR). To induce neural progenitor differentiation, mTeSR medium was replaced with N2 medium: DMEM/F12 (Gibco, Cat. #11320-33), 2% B-27 supplement (Gibco, Cat. #17504044), 1% N-2 supplement (Gibco, Cat. #17502-048), 1% MEM Non-Essential Amino Acids (Gibco, Cat. #11140-050), 1% penicillin/streptomycin (Gibco, Cat. #15140148), 0.1% β-mercaptoethanol (Gibco, Cat. #21985-023), cells were supplemented daily with dual SMAD inhibitors 10 μM SB-431542 (Milteny Biotec, Cat. #130-106-543) and 0.1 µM LDN-193189 (Stemgent, Cat. #04-0074) for 10 days^51^. Fresh medium was daily replaced. On day 11, neural progenitors were detached using Accutase (Gibco, Cat. #A11105-01), dissociated single cells were then seeded onto coverslips coated with Poly-l-ornithine (Sigma, Cat. #P4957) and 5 mg/ml Laminin (Thermofisher, Cat. #23017015) in 18 mm cover glass in the presence of basic fibroblast growth factor (bFGF, 20 ng/ml; R&D, Cat. #233-FB-01M) for 12 h. To induce neuronal differentiation, N2 medium was replaced with neurobasal medium (Gibco, Cat. #A35829-01) containing 2% B-27, 1% N-2, 1% penicillin/streptomycin, 0.025% Insulin (Sigma, Cat. #I9278), 10 ng/ml BDNF (Lonza-Peprotech, Cat. #450-02-50), 0.2 µg/ml l-Ascorbic acid (Sigma, Cat. #A4544) and 0.1 mM cAMP (Sigma, Cat. #D0627). After 2 weeks of differentiation, neurons were fixed with 4% paraformaldehyde (PFA) (Thermofisher, Cat. #ALF043368.9 M) for 10 min at room temperature (RT) and immunostained with neuronal markers.

### Immunohistochemistry

Tissue processing was performed as described in ref. ^52^ and immunohistochemistry (IHC) was performed as described in ref. ^53^. In brief, organoids were fixed in 4% PFA for 60 min at RT, followed by washing three times with 1× phosphate buffer saline (PBS) for 10 min at RT before sectioning. Fixed organoids were then immersed in 30% sucrose in PBS at 4 °C. Sunk organoids were then embedded in a solution containing at 3:2 ratio Optimal Cutting Temperature (O.C.T) and 30% sucrose on dry ice. Embedded tissues were then sectioned serially to the 14-µM thickness and collected onto Superfrost slides (Thermo Scientific, cat. #SF41296). For IHC, sectioned samples were washed three times for 10 min at RT before blocking for 1 h with a solution containing 3% bovine serum albumin (Sigma, Cat. A9418-50G) and 0.1% Triton X-100 in PBS. Primary antibodies were added overnight at 4 °C before washing three times with PBS for 10 min each at RT. For immunocytochemistry^54^, cells were fixed with 4% PFA in 1× PBS for 10 min at RT. Fixed cells were then washed three times with 1× PBS at RT before blocking and adding the primary antibody as stated above. Tissues and cells were then incubated with respective secondary antibodies for 1 h at RT before mounting and imaging. All tissues and cells were counterstained with Hoechst 33342 (Invitrogen, Cat. #H3570). All images were acquired using confocal microscopy (Leica TCS SP8) based in SBMS Imaging Facilities based at the University of Queensland. The primary antibodies used in this study are listed in Supplementary Table 5. Alexa-488, Alexa-546, and Alexa-633-conjugated secondary antibodies were obtained from Jackson ImmunoResearch Laboratory.

### Senescence-associated β-galactosidase assay (SA-β-Gal)

2D neurons were fixed with 4% PFA in PBS, pH 7.4, for 10 min at RT and incubated at 37 °C overnight in staining solution as previously described in ref. ^55^. Brain organoids were fixed in 4% PFA in PBS, pH 7.4, for 1 h at RT and washed with PBS for 10 min at RT before allowing to sink in 30% sucrose at 4 °C. Once sunk, organoids were immediately embedded in OCT (Agar Scientific, cat. #AGR1180), cryosectioned at 14 μm, and incubated at 37 °C overnight in staining solution. Staining was evident in 2–4 h and maximal in 12–16 h. Samples were examined under phase-contrast microscopy.

### Telomere FISH assay

2D neurons were fixed in 4% PFA in PBS for 10 min. Cells were washed in PBS, dehydrated in ethanol series (70, 95, 100%), and air-dried. Coverslips were denatured for 5 min at 80 °C in hybridization mix (70% formamide, 10 mM Tris–HCl (pH 7.2), and 0.5% Roche blocking solution) containing telomeric PNA-(CCCTAA)_3_ probe. After denaturation, hybridization was continued for 2 h at RT in a dark humified chamber. Coverslips were washed twice for 15 min each with 70% formamide, 10 mM Tris–HCl (pH 7.2), and 0.1% BSA, and followed by three washes for 5 min each with 0.15 M NaCl, 0.1 M Tris–HCl (pH 7.2), and 0.08% Tween-20. Nuclei were stained with DAPI (0.5 μg/ml) in PBS and slides were mounted in mowiol solution (Calbiochem). Images were obtained with a Leica TCS SP8 MP confocal laser microscope by the acquisition of optical *z*-sections at different levels along the optical axis. Telomere length was analyzed by quantification of telomeric signal fluorescence intensities by the imaging software CellProfiler. Comparative immunofluorescence analyses were performed in parallel with identical acquisition parameters.

### qRT-PCR

Total RNA was isolated from organoids as described previously^56^. For qPCR, SYBR Green (Applied Biosystem, Cat. #A25742) was used. 1 µg of RNA was utilized to generate the cDNA using the First Strand cDNA Synthesis Kit (Thermo Scientific, Cat. #K1612). PCR standard reaction conditions were set according to the manufacturer’s instructions. PCR primers were designed using the NCBI free online system. All experiments were performed in biological duplicates or triplicates for each sample analyzed. Expression values were normalized against the GAPDH expression value of each sample, means and standard deviations were calculated and plotted using the GraphPad Prism 8.3.1^®^ and Sigma Plot 13.0^®^ software. Primers are listed in Supplementary Table 6.

### Western blot

Western blotting was performed as described previously^57^. In brief, organoids were collected and lysed in Pierce^TM^ RIPA Buffer (ThermoFisher Scientific, Cat. #89900), and a cocktail of protease and phosphatase inhibitors (Roche). Organoids were then sonicated, and protein concentration was quantified using Pierce^TM^ bicinchoninic acid (BCA) protein assay kit (ThermoFisher Scientific, Cat. #23227) according to the manufacturer’s instructions. Samples were then heated for 10 min at 100 °C before loading. An equal amount of proteins was loaded and separated using Mini-PROTEAN TGX Stain-Free-Gels (Bio-Rad, Cat. #4568044), separated proteins were then transferred onto iBlot 2 PVDF Mini Stacks (Invitrogen, Cat. #IB24002). The membrane was then blocked with 5% Skim Milk in TBS-T (20 mM Tris–HCl, pH 7.6, 136 mM NaCl, and 0.1% Tween-20) for 1 h at RT, followed by primary antibody incubation diluted in 5% BSA for 12 h at 4 °C. The primary antibodies used in this experiment are listed in Supplementary Table 5. The membrane was then washed three times with 1× TBST for 10 min each at RT before incubation with secondary antibody diluted 1:5000 in 5% Skim Milk in 1× TBST for 1 h at RT. The membrane was washed again three times with 1× TBST for 10 min each at RT before visualization with Clarity Western ECL Substrate (Bio-Rad, Cat. #170-5060).

### RNA-sequencing

A total amount of 1 μg RNA per sample was used as input material for the RNA sample preparations. Sequencing libraries were generated using NEBNext® UltraTM RNA Library Prep Kit for Illumina® (NEB, USA) following the manufacturer’s recommendations and index codes were added to attribute sequences to each sample. Briefly, mRNA was purified from total RNA using poly-T oligo-attached magnetic beads. Fragmentation was carried out using divalent cations under elevated temperature in NEBNext First Strand Synthesis Reaction Buffer (5X). First-strand cDNA was synthesized using random hexamer primer and M-MuLV Reverse Transcriptase (RNase H-). Second strand cDNA synthesis was subsequently performed using DNA Polymerase I and RNase H. Remaining overhangs were converted into blunt ends via exonuclease/polymerase activities. After adenylation of 3′ ends of DNA fragments, NEBNext Adaptor with hairpin loop structure was ligated to prepare for hybridization. To select cDNA fragments of preferentially 150–200 bp in length, the library fragments were purified with AMPure XP system (Beckman Coulter, Beverly, USA). Then 3 μl USER Enzyme (NEB, USA) was used with size-selected, adaptor-ligated cDNA at 37 °C for 15 min followed by 5 min at 95 °C before PCR. Then PCR was performed with Phusion High-Fidelity DNA polymerase, Universal PCR primers, and Index (X) Primer. At last, PCR products were purified (AMPure XP system) and library quality was assessed on the Agilent Bioanalyzer 2100 system. Raw data (raw reads) of FASTQ format were firstly processed through fastp. In this step, clean data (clean reads) were obtained by removing reads containing adapter and poly-N sequences and reads with low quality from raw data. At the same time, Q20, Q30, and GC content of the clean data were calculated. All the downstream analyses were based on clean data with high quality. Reference genome and gene model annotation files were downloaded from the genome website browser (NCBI/UCSC/Ensembl) directly. Paired-end clean reads were mapped to the reference genome using HISAT2 software. HISAT2 uses a large set of small GFM indexes that collectively cover the whole genome. These small indexes (called local indexes), combined with several alignment strategies, enable rapid and accurate alignment of sequencing reads. Stringtie was used to assemble the set of transcript isoforms of each bam file obtained in the mapping step. gffcompare can compare Strintie assemblies to reference annotation files and help sort out new genes from known ones. Featurecounts were used to count the read numbers mapped of each gene, including known and new genes. And then RPKM of each gene was calculated based on the length of the gene and reads count mapped to this gene.

### Bioinformatics analysis

RNA-sequencing normalized data GSE110006^18^, GSE110006^19^, and GSE97881^20^ were downloaded. H9-ESCs group was used as a control to calculate the fold change of genes expression across different times of organoids culture. Lists of senescence increased and decreased genes were isolated from these RNA-seq databases according to a recent study^21^. Each database was normalized as explained before^18–20^. Normalized mRNA expression was further normalized between 0 (lowest FPKM) to 1 (highest FPKM) to generate the heatmap (Fig. 1i). Week 1 was considered a control group to calculate the fold change of normalized FPKM value (Fig. 1h).

### Statistical and image analysis

Normally distributed data were expressed as the mean ± standard deviation of the mean of independent experiments. The median ± standard deviation was used to express the non-normally distributed data. For organoid size measurements, organoids were imaged over time using the Olympus CKX53 microscope and an Olympus SC50 camera. The Olympus cellSens Entry software was used to generate the scale bar and corresponds to the objective lens. Images of organoids were exported to ImageJ to measure the diameter of individual organoids. The shortest distance across the organoid was measured and assigned as organoid diameter. For neurites diameter measurement, confocal images with scale bar were exported to ImageJ, lines were manually drawn onto the confocal images and the pixel length of the lines was used to calculate the average neurite diameter, calibrated against a given scale. The sample size was determined using power analysis. The number of biological replicates, as well as the sample size, are indicated in the figure legends. When comparing two groups, we used the Student’s *t*-test. A one-way or two-way ANOVA was used for the comparison of multiple groups, followed by Tukey’s post-hoc analysis for comparisons to a single control. Statistical analysis was performed using Sigma Plot 13^®^ software. Minimal statistical significance was defined at *P* < 0.05.

### Reporting summary

Further information on research design is available in the Nature Research Reporting Summary linked to this article.

## Supplementary information

Supplementary Information

Supplementary Data 1

Supplementary Data 2

Reporting Summary

## Data Availability

The data that support the findings of this study are available from the corresponding author upon reasonable request. Bulk RNA-sequence data from brain organoids that support the findings of this study have been deposited in the GEO-NCBI with the primary accession code GSE171719.
