# Supplementary material for: Klotho inhibits neuronal senescence in human brain organoids
Source: NPJ Aging Mech Dis. 2021 Aug 2;7:18. doi: 10.1038/s41514-021-00070-x (PMC8329278; doi:10.1038/s41514-021-00070-x)

Klotho inhibits neuronal senescence in human brain organoids

Mohammed R. Shaker[1*], Julio Aguado[1], Harman Kaur Chaggar[1], and Ernst J. Wolvetang[1*]

[1]Australian Institute for Bioengineering and Nanotechnology, The University of Queensland, Queensland 4072, Australia.

[*]Correspondence to be addressed to:
[*]Mohammed R. Shaker, PhD
Research Fellow
Stem Cell Engineering group
The Australian Institute for Bioengineering and Nanotechnology (AIBN)
Level 4 west, Building 75, Corner College and Cooper Rds, St Lucia campus.
The University of Queensland
Brisbane Qld 4072 Australia
T +61 7 3346 3835 M +61 434 449 446
m.shaker@uq.edu.au

*Professor Ernst J. Wolvetang, PhD
Senior Group Leader
Stem Cell Engineering group
The Australian Institute for Bioengineering and Nanotechnology (AIBN)
Level 4 west, Building 75, Corner College and Cooper Rds, St Lucia campus.
The University of Queensland
Brisbane Qld 4072 Australia
T +61 7 3346 3894 M +61 466 655 536
e.wolvetang@uq.edu.au

Supplementary Information

Supplementary Figures

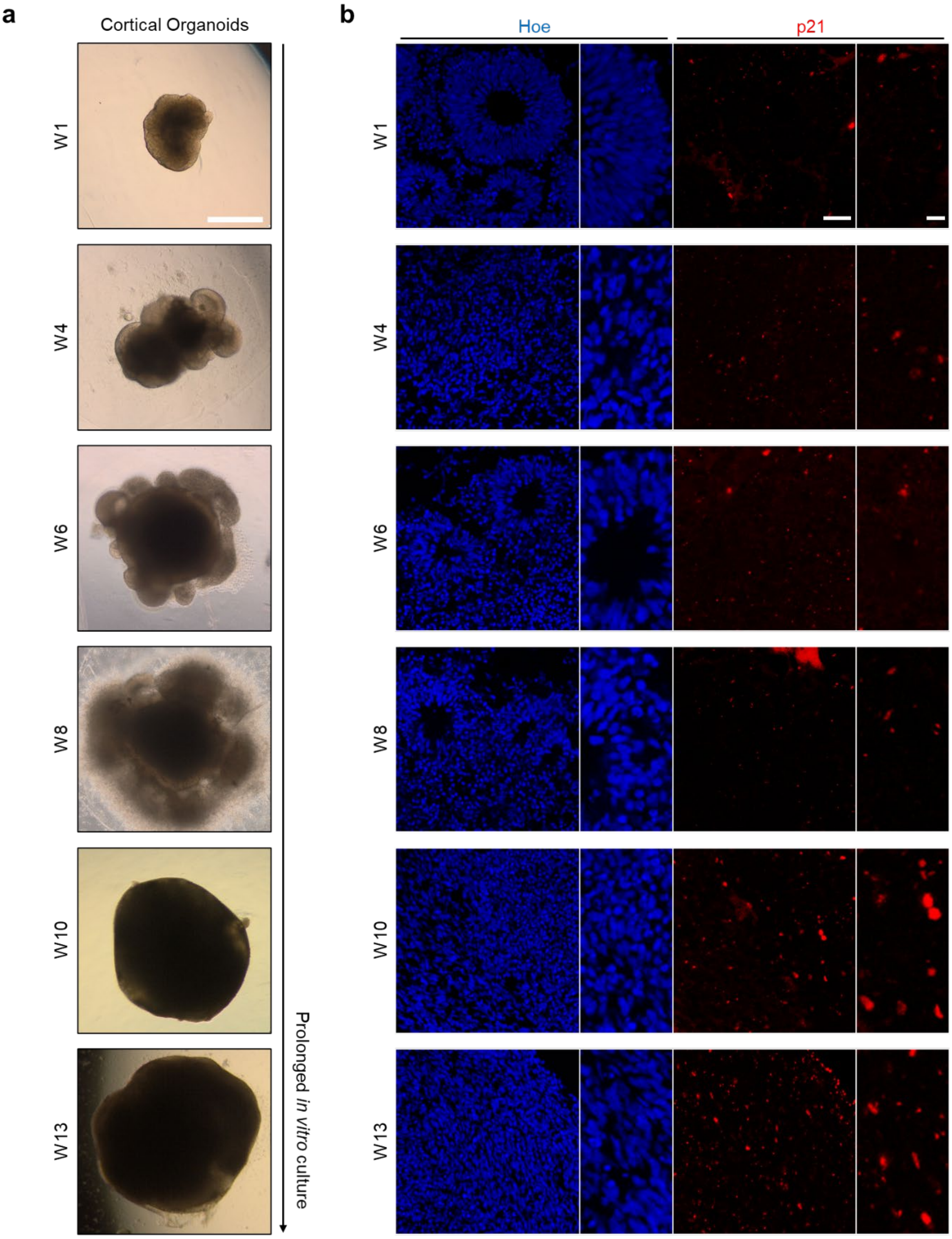

Supplementary Fig. 1 Culture of cortical brain organoids over extended period of time.

(**a**) Representative images of cortical organoids derived from human H9-ESCs cultured over 13 weeks *in vitro*. Scale bar = 600 µm.

(**b**) Representative images of sections of different ages of human cortical brain organoids derived from human H9-ESCs. Sections were stained with p21 antibody (Red) and counterstained with Hoechst 33342 (Blue). Scale bar = 65 µm. W is week.

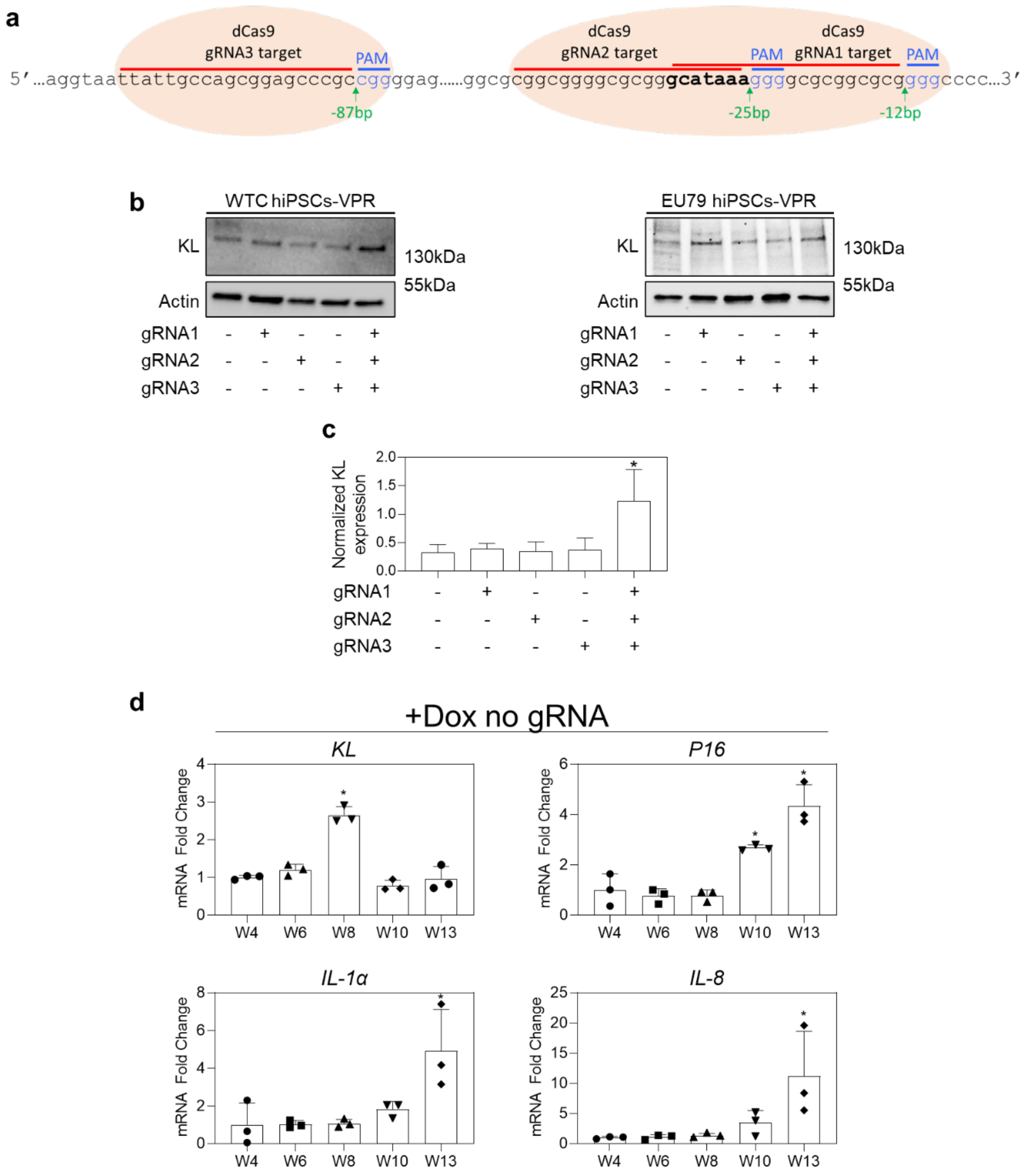

Supplementary Fig. 2 Sequence of gRNAs and KL protein expression in human iPSCs with VPR.

(**a**) Schematic representation of the location of the three gRNAs targeting sites upstream of the *KL* promoter. Red lines highlight the sequence of each gRNA.

(**b**) Western blots showing the protein levels of KL in human iPSCs using the WTC line (Left) and EU79 line (Right) transduced with gRNA1, gRNA2 or gRNA3 lentivirus or transduced

with all three gRNAs. Transduced human iPSCs were treated with dox for 7 days. All blots derive from the same experiment and processed in parallel.

(**c**) Quantification of KL level from data in (**b**). Data are shown as mean ± standard deviation. The number of independent experiments = 3. *$P < 0.05$ via One Way ANOVA.

(**d**) Fold change of mRNA levels of KL, P16, IL-1A and IL-8. All values were normalized to GAPDH levels of their respective samples and expressed relative to W4 cortical brain organoids values to obtain the fold change. Data are shown as mean ± standard deviation; Number of independent experiments = 3, total number of analyzed organoids = 72; *$p < 0.05$ via One Way ANOVA. W is week.

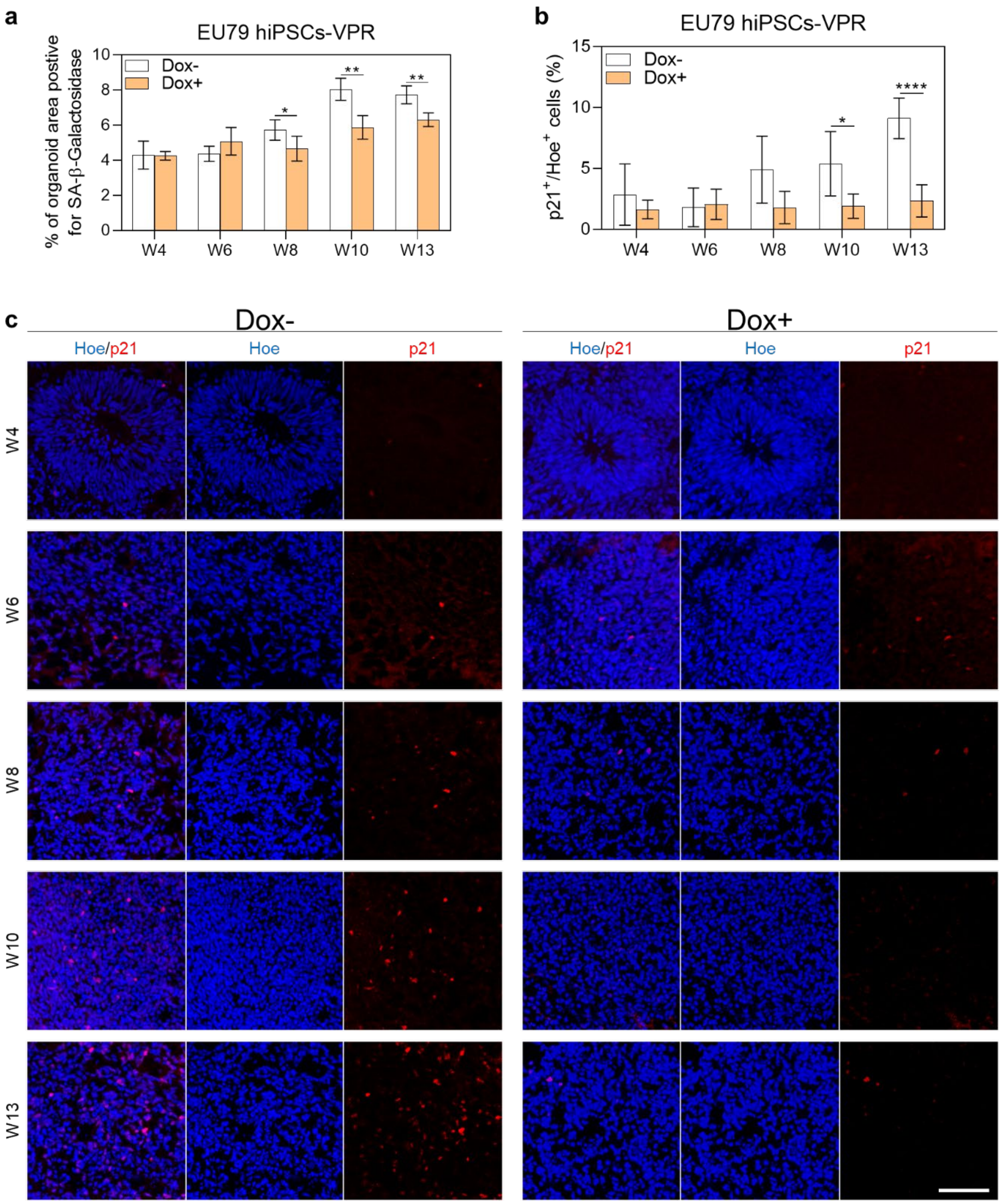

Supplementary Fig. 3 Senescence associated markers in cortical brain organoids derived from iPSCs.

(a) Quantification of the percentage of SA-β-gal area normalized to the total area of each individual organoid derived from human dCas9-VPR iPSCs (EU79 line). Data are presented

as mean ± standard deviation. **P<0.001 via One Way ANOVA. Number of independent experiments = 3. Total number of analyzed organoids = 72. W is week.

(**b**) Quantification of the percentage of total p21^{+} cells relative to the total number of cells per organoids derived from human dCas9-VPR iPSCs (EU79 line). Data are presented as mean ± standard error mean. *P<0.05, ****P<0.0001 via One Way ANOVA. Number of independent experiments N= 3. Total number of analyzed organoids = 72. W is week.

(**c**) Representative images of sections of human cortical brain organoids of different ages derived from human dCas9-VPR iPSCs (WTC line) transduced with 3 gRNAs and treated with dox from week 4 to week 13. Dox- represents organoids without dox treatment. Tissues were stained with p21 antibody (Red). All sections were counterstained with Hoechst 33342 (Blue). Scale bar = 100 µm. W is week.

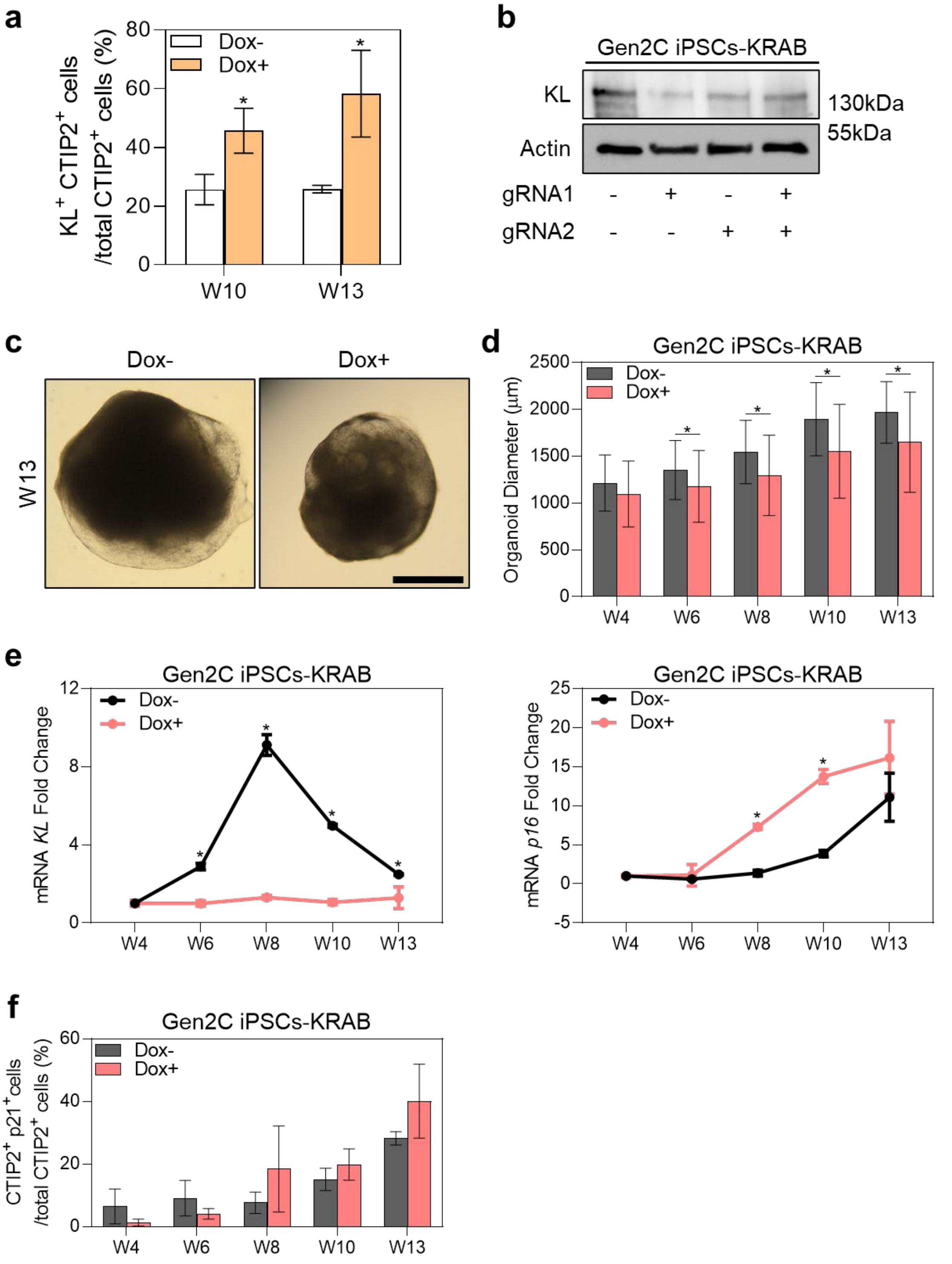

Supplementary Fig. 4 Generation and characterization of cellular senescence using Gen2C iPSCs-KRAB line.

(**a**) Percentage of CTIP2+ KL+ neurons relative to total CTIP2+ neurons per organoid in organoids with (Dox+) and without *KL* induction (Dox-) derived from human dCas9-VPR

iPSCs (WTC line). Data are presented as mean ± standard deviation. *$p < 0.05$ via Student's t test. W is week. Number of independent experiments N= 3. Total number of analyzed organoids = 24. W is week.

(**b**) Western blots showing the protein levels of KL in pooled human iPSCs using the Gen2C line (Left) transduced with gRNA1 or gRNA2 lentivirus or transduced with the two gRNAs. Transduced human iPSCs were treated with dox for 7 days. All blots derive from the same experiment and processed in parallel.

(**c**) Representative images of cortical organoids derived from human Gen2C iPSCs-KRAB cultured for 13 weeks *in vitro*. Scale bar = 600 µm.

(d) Measurement of the diameter of organoids derived from human Gen2C iPSCs-KRAB line with and without dox treatment from week 4 to week 13. Data are the mean ± standard deviation. *$P<0.05$ via t-test. Number of independent experiments N= 4. Total number of analyzed organoids = 307.

(**e**) qRT-PCR of *KL* and *P16* at different stages of *in vitro* culture of human cortical brain organoids derived from human Gen2C iPSCs-KRAB following daily treatment with dox from week 4 to week 13. Dox- represents organoids without dox treatment. All values were normalized to GAPDH levels of their respective samples, normalized data were further normalized to W4 to obtain the fold change value. Data are shown as mean ± standard error mean; Number of independent experiments N= 3. Total number of analyzed organoids = 60; * $P<0.05$ via One Way ANOVA. W is week.

(**f**) Percentage of CTIP2+ neurons expressing p21 relative to total cells per organoid in organoids with (Dox+) and without *KL* induction (Dox-). Data are presented as mean ± standard error mean. Number of independent experiments N= 3. Total number of analyzed organoids = 90. W is week.

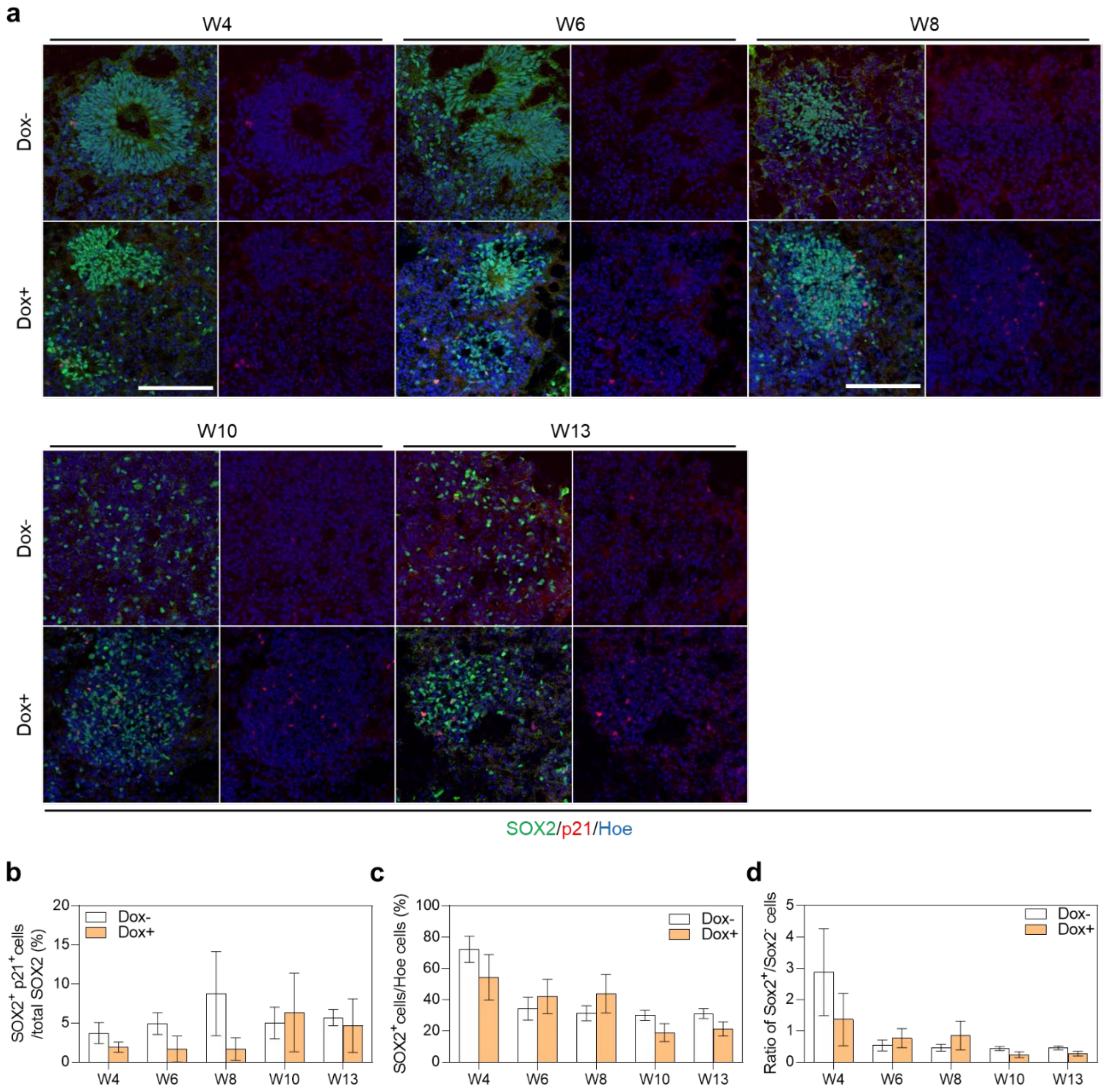

Supplementary Fig. 5 Distribution of neural progenitors in cortical brain organoids with and without *KL* overexpression.

(**a**) 4 to 13 weeks cortical brain organoids sectioned and immunostained with SOX2 (Green) and p21 (Red) antibodies. All sections were counterstained with Hoechst 33342 (Blue). Dox- indicates cortical organoids without daily dox treatment. Dox+ indicates cortical organoids with daily dox treatment. Scale bar = 100 μm.

(**b**) Percentage of SOX2+ cells expressing p21 relative to total SOX2+ cells per organoid in organoids with (Dox+) and without *KL* overexpression (Dox-). Data are presented as mean ± standard error mean. Number of independent experiments N= 4. Total number of analyzed organoids = 80. W is week.

(**c**) Percentage of SOX2+ cells relative to total cells per organoid in organoids with (Dox+) and without *KL* overexpression (Dox-). Data are presented as mean ± standard deviation. Number of independent experiments N= 4. Total number of analyzed organoids = 80. W is week.

(**d**) Ration of SOX2+ cells over SOX2- cells per organoid in organoids with (Dox+) and without *KL* overexpression (Dox-). Data are presented as mean ± standard deviation. Number of independent experiments N= 4. Total number of analyzed organoids = 80. W is week.

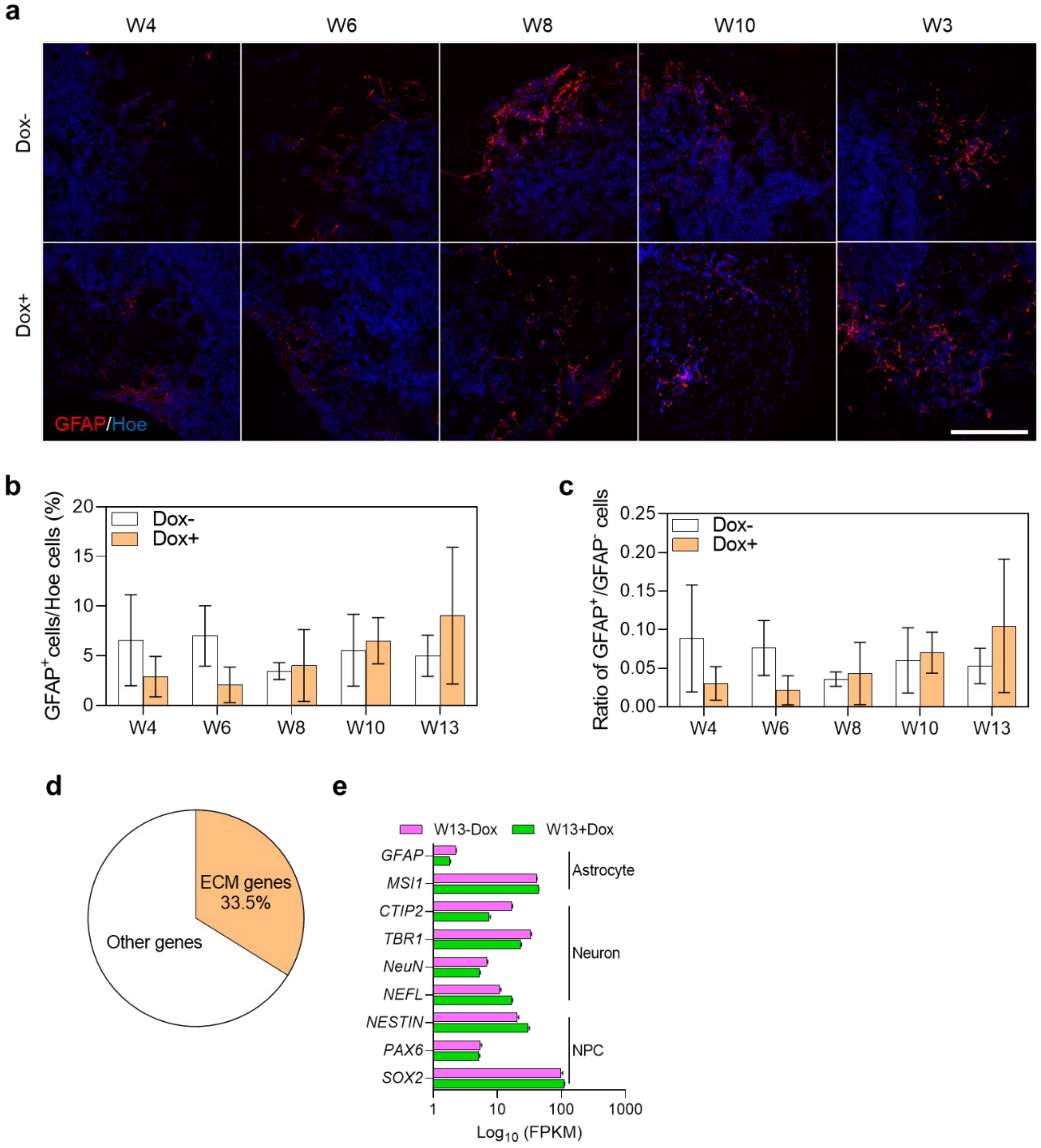

Supplementary Fig. 6 Distribution of astrocytes in cortical brain organoids with and without *KL* overexpression.

(**a**) 4 to 13 weeks cortical brain organoids derived from human dCas9-VPR iPSCs (EU79 line) sectioned and immunostained with GFAP antibody (Red). All sections were counterstained with Hoechst 33342 (Blue). Dox- indicates cortical organoids without daily dox treatment. Dox+ indicates cortical organoids with daily dox treatment. Scale bar = 100 μm.

(**b**) Percentage of GFAP+ cells relative to total cells per organoid in organoids with (Dox+) and without *KL* overexpression (Dox-) derived from human dCas9-VPR iPSCs (EU79 line).

Data are presented as mean ± standard deviation. Number of independent experiments N= 3. Total number of analyzed organoids = 72. W is week.

(**c**) Ratio of GFAP+ cells over GFAP- cells per organoid in organoids with (Dox+) and without *KL* overexpression (Dox-) derived from human dCas9-VPR iPSCs (EU79 line). Data are presented as mean ± standard deviation. Number of independent experiments N= 4. Total number of analyzed organoids = 72. W is week.

(**d**) Pie chart shows the percentage of ECM genes among the top 10% of upregulated genes in cortical brain organoids at week 13 upon overexpression of *KL* compared to the control group.

(**e**) Relative expression of neural cell types markers mRNA obtained from RNA-seq data of cortical brain organoids at week 13 with (Dox+) and without (Dox-) overexpression of KL. Data are presented as mean ± standard deviation. NPC indicates neural progenitor cells.

Supplementary Tables

Supplementary Table 1. List of Significantly Upregulated Genes in Cortical Brain Organoids.

- ❖ Table is provided in supplementary excel file.

Supplementary Table 2. List of Significantly Downregulated Genes in Cortical Brain Organoids.

- ❖ Table is provided in supplementary excel file.

Supplementary Table 3. List of Genes in KEGG Enrichment Analysis Among Downregulated Genes in Cortical Brain Organoids.

Description	pvalue	padj	geneName
Dopaminergic synapse	3.67E-11	1.12E-08	GRIA1/GRIA2/GRIA3 /PRKACB/PPP2R2B/ PLCB1/GRIN2B/ CAMK2A/PRKCA /GNAI1/KIF5C/KIF5A /PPP3CB/CACNA1B /GNAQ/PPP2R3A /PPP1R1B/DRD2 /MAPK11/PPP2R5C /PPP3CA/GSK3B /ADCY5/CALM1/PPP2R2C /GNAO1/MAPK8/PRKCG /CAMK2B/CACNA1A/GNG2 /KIF5B/PPP1CA/CREB5 /CALM2/PPP2CA/KCNJ3 /CALY/PPP2R5E/CREB1 /PPP2R5B/GRIN2A /CACNA1D/MAPK9
Glutamatergic synapse	5.11E-10	7.80E-08	GRIK3/GRIA1/GRIA2 /ADCY1/SLC1A2/SLC17A7 /DLGAP1/GRIA3/PRKACB /SLC38A1/PLCB1/GRIN2B /GRIN1/PRKCA/SHANK2 /SLC1A3/GNAI1/PPP3CB /GNAQ/GRK3/GRIK2 /PPP3CA/ADCY5/GNAO1 /GRIK5/PRKCG/SLC1A1 /GRM3/CACNA1A/GNG2 /MAPK1/GRM2/HOMER1 /SHANK1/KCNJ3/GRM5 /GRIN2A/CACNA1D/SLC1A6
Nicotine addiction	2.64E-09	2.69E-07	GRIA1/GRIA2/GABRB3 /SLC17A7/GRIA3/GABRA5 /GRIN2B/GRIN1/GABRG2

			/CACNA1B/GABRA4 /GABRB2/CHRNB2/CHRNA4 /GABRA2/GABRA3/CACNA1A /GRIN2A/GABRG3
Synaptic vesicle cycle	6.06E-09	4.62E-07	CPLX2/SLC1A2/SLC6A1 /SLC17A7/SLC6A13/SNAP25 /SYT1/UNC13B/SLC1A3 /UNC13A/CACNA1B/STXBP1 /ATP6V1B2/SLC6A12 /ATP6V1C1/SLC1A1/SLC6A7 /STX3/CACNA1A/CLTB /ATP6V1D/SLC6A11/ATP6V0E2 /CLTC/DNM3/STX1B /AP2A2/CPLX1/ATP6V0A2 /SLC1A6
Long-term potentiation	3.48E-08	2.12E-06	GRIA1/GRIA2/ADCY1 /PRKACB/PLCB1/GRIN2B /CAMK2A/CAMK4/GRIN1 /PRKCA/PPP3CB/KRAS /GNAQ/PPP3CA/CALM1 /PRKCG/CAMK2B/RPS6KA2 /EP300/PPP1CA/MAPK1 /CALM2/NRAS/RAF1/GRM5 /GRIN2A/RPS6KA6
Adrenergic signaling in cardiomyocytes	1.32E-07	6.73E-06	ADCY1/PRKACB/PPP2R2B /PLCB1/CAMK2A/CACNG8 /SLC8A1/ATP2B2/PRKCA /GNAI1/GNAQ/PPP2R3A /ACTC1/MAPK11/PPP2R5C /ADCY5/ATP2B3/CALM1 /PPP2R2C/SLC8A3/RYR2 /CAMK2B/ATP1B3/ATP2A2 /PPP1CA/CREB5/MAPK1 /CALM2/PPP2CA/CACNB4 /ATP2A3/CACNG4/SCN5A /PPP2R5E/ATP2B1/CREB1 /PPP2R5B/CACNB2/CACNA2D1 /CACNA1D
Circadian entrainment	3.36E-07	1.47E-05	GRIA1/GRIA2/GUCY1A1 /ADCY1/GRIA3/PRKACB /CACNA1H/PLCB1/GRIN2B /CAMK2A/GUCY1B1/GRIN1 /PRKCA/GNAI1/ADCYAP1R1 /GNAQ/ADCY5/CALM1/GNAO1 /RYR2/PRKCG/CAMK2B /GUCY1A2/RYR3/GNG2 /MAPK1/CALM2/KCNJ3 /CREB1/GRIN2A/CACNA1D
Amphetamine addiction	8.46E-07	3.17E-05	GRIA1/GRIA2/GRIA3/PRKACB /GRIN2B/CAMK2A/CAMK4 /GRIN1/PRKCA/PPP3CB

			/PPP1R1B/PPP3CA/ADCY5 /CALM1/PRKCG/CAMK2B /PPP1CA/CREB5/CALM2 /CREB1/GRIN2A/PDYN /CACNA1D
ErbB signaling pathway	9.36E-07	3.17E-05	SHC3/CAMK2A/PRKCA /PIK3R3/PAK3/KRAS /MTOR/ABL2/PIK3CB /GSK3B/PAK5/MAPK8 /PRKCG/CAMK2B/BTC /MAP2K4/GRB2/SOS1 /RPS6KB1/PAK6/PAK2 /MAPK1/SHC2/NRAS /CBL/SOS2/RAF1/MAPK9
Morphine addiction	1.17E-06	3.53E-05	ADCY1/PDE1A/GABRB3 /GABRA5/PRKACB/PRKCA /GABRG2/GNAI1/CACNA1B /GABRA4/GABRB2/GRK3 /GABBR2/PDE2A/GABRA2 /GABRA3/ADCY5/GNAO1 /PRKCG/CACNA1A/PDE7A /PDE4A/GNG2/GRK4/PDE8B /KCNJ3/ADORA1/GABRG3

Supplementary Table 4. List of Upregulated Genes Identified in Volcano Plot Analysis.

Gene	Log2(fold change)	-LOG10(adj.p-value)
PTGER4	2.003185	1.529403
APOA1	2.006644	1.805866
DGKK	2.011582	2.03423
CCDC3	2.013984	146.6715
CBLN2	2.016466	8.78188
AC004947.2	2.018047	2.216099
CTXND1	2.019426	5.926072
STAT4	2.026262	3.440096
GRID2	2.029411	22.7889
NAALAD2	2.032417	4.192834
ITM2A	2.032558	60.64617
AC044849.1	2.045294	2.602816
DLX3	2.046106	1.135019
THBS1	2.048309	53.07926
SLC5A7	2.048805	1.623606
KRT8P36	2.053203	2.117934
MME	2.053419	26.95961
AC002454.1	2.069741	1.170732
LMX1B	2.071374	12.54582

TWIST1	2.074821	80.89032
PKHD1	2.075951	1.472036
WNT11	2.07903	50.46032
A4GALT	2.07915	3.243211
MATN2	2.087772	100.2532
SPP1	2.088254	2.761589
TBX2-AS1	2.090258	1.376228
ACVRL1	2.095847	20.83047
MEIS1	2.097045	47.75537
PRND	2.097725	2.15326
POSTN	2.101808	0
GPC3	2.102395	132.4085
MPZ	2.102701	56.25451
ABCC9	2.106328	5.625322
EBF1	2.108719	45.12137
AC092957.1	2.108913	4.425415
ZIC1	2.109439	0
SYPL2	2.110836	1.837154
HIST1H2BF	2.122334	3.171309
ALX1	2.136324	17.24892
AL021395.1	2.147202	5.734913
CNTNAP4	2.148036	15.24772
ACTN3	2.149781	1.653381
AL138885.3	2.14988	1.674544
LHX1	2.152788	2.287274
ADAM33	2.155541	145.6105
SECTM1	2.161671	37.46275
IRX1	2.174707	55.23354
COBL	2.183189	14.00776
COL23A1	2.187916	31.14175
C3	2.19066	2.663657
ALX4	2.199032	71.95819
AQP7P1	2.199268	2.978788
TMEM229A	2.202328	3.239007
PBX3	2.204681	141.5151
MRGPRF	2.211146	15.22522
ZIC4	2.212876	111.8858
ENPP2	2.213994	157.597
FGF1	2.214415	17.17259
CCDC68	2.220858	1.066831
KCNH1	2.222002	2.009176
ST8SIA6	2.237542	1.055032
PAX5	2.23922	3.609094
TMEM119	2.251018	59.27742

KLK10	2.252067	2.704812
WNT6	2.255491	1.454825
ANGPTL6	2.255492	13.51445
GBP2	2.255611	2.315211
LINC01305	2.256618	11.60783
PTGER2	2.279292	4.176368
MEOX2	2.2837	1.92642
DPT	2.297461	7.567251
CU633906.2	2.300218	3.635864
ZNF295-AS1	2.30773	1.081444
ASPN	2.314672	66.73812
PACERR	2.324136	1.159743
C22orf42	2.339445	8.266155
ADAMTS18	2.341846	3.500182
SPARCL1	2.345599	269.8842
AL121658.1	2.346494	1.118346
KL	2.363247	1.702236
AC007424.1	2.370641	2.20989
IRX4	2.37307	2.859522
KLK1	2.378738	1.179287
ESPN	2.38396	76.54193
HOXB3	2.384284	1.741827
LTBR	2.385878	2.607258
OMD	2.386746	5.279868
FXYD3	2.391403	2.496628
ADGRG2	2.396157	4.60474
KIAA0040	2.400919	5.587885
ELN	2.406786	0
BMP3	2.412945	4.455846
PAX1	2.414362	27.61607
TRDN	2.415513	3.816615
RUBCNL	2.417556	4.14425
SP9	2.418779	3.568841
TRHR	2.420516	2.109445
CNTN5	2.426226	7.146615
TWIST2	2.4342	52.44748
LRRC77P	2.438959	1.282975
OSR2	2.442757	23.32739
COL20A1	2.450201	137.9525
AC004080.1	2.452978	2.108986
RNFT1P3	2.460683	1.649412
GBA	2.464006	2.429461
DMRT2	2.471513	4.224417
AL096865.1	2.477731	1.99896

IGSF10	2.5198	24.95051
AC010247.2	2.522739	2.156801
MC4R	2.533671	3.060648
SCN7A	2.56011	3.237053
KRT17	2.562204	5.135346
HOXB2	2.577647	2.044497
AC008013.2	2.599521	1.245458
C10orf90	2.61084	9.300228
COL19A1	2.615819	9.975325
IRX6	2.616978	4.435649
WNT10A	2.620311	4.773017
NFATC2	2.625752	32.91669
IL18R1	2.63305	1.290554
ASPA	2.660301	9.543068
MAB21L2	2.683469	62.47661
TNMD	2.686406	3.944783
ADCYAP1	2.697377	32.66174
DIO3	2.699602	10.09878
COL8A2	2.708533	103.4633
TAC1	2.730464	41.04721
FZD10-AS1	2.739371	13.56075
OLFML2A	2.752309	0
KLHL1	2.761856	17.23304
RIPOR3	2.767697	15.31652
INSC	2.773069	5.745374
GAD1	2.786662	239.4579
ERBB3	2.796258	70.28175
TMEM71	2.823358	2.712511
AL031710.2	2.828086	1.586334
EPS8L1	2.839986	67.57751
RNF144B	2.857322	1.441421
SLC35F4	2.880606	2.080509
NXPH1	2.883485	22.43763
COMP	2.888105	3.806943
GSC	2.901319	2.596951
MAL	2.904552	33.86559
SKOR1	2.911396	69.68423
FOXB1	2.92277	5.265545
EN2	2.927234	35.603
PTGS2	2.945637	9.400185
DIO3OS	2.959065	30.58184
GFRA1	2.966672	179.305
CDH19	2.990236	36.47756
SOX10	2.994538	184.938

AP002004.1	3.013915	4.841471
FOXL2NB	3.022556	13.85207
SEMA3G	3.022947	80.47486
FOXD3	3.026605	29.60544
THBD	3.038513	21.66677
OLIG3	3.061802	3.412568
AL139383.1	3.088755	1.549252
KIFC1	3.10094	1.55498
GPR17	3.114576	33.13991
ATP10B	3.131057	1.515388
C1QL4	3.185958	42.20742
FGFBP2	3.198752	15.46885
SHOX2	3.201671	20.68496
GFRA3	3.213172	111.4357
LINC01198	3.232554	2.74486
IRX2	3.233769	45.73781
PRL	3.25167	3.615488
SIX2	3.261049	23.99905
CEACAM21	3.261718	1.853874
HES2	3.266742	7.152314
FOXD3-AS1	3.272743	16.38305
VGLL3	3.315276	108.4635
PAX3	3.317355	58.80256
AP000894.2	3.330336	17.76407
HPSE2	3.352021	6.545891
MRGPRF-AS1	3.354931	2.323295
ADH1B	3.382823	11.29585
IRX3	3.418266	21.12307
ACAN	3.429121	37.19772
AC087477.2	3.438388	5.768167
FZD10	3.457509	24.22117
PITX2	3.469119	12.94725
AL353747.4	3.487959	3.606024
PTPRT	3.511139	52.53953
FOXL2	3.518217	10.37896
DBX1	3.552064	6.188897
BIRC3	3.564971	3.67744
PKHD1L1	3.596489	2.31059
PI15	3.619793	56.31271
MS4A6A	3.642093	2.44636
AC091980.2	3.688359	3.389333
GSDMD	3.693122	5.404883
CASP12	3.726723	1.77794
GRAP2	3.729151	1.716539

ONECUT3	3.744749	6.251846
APOBEC3D	3.762455	1.82181
IRX5	3.764365	65.72968
C5orf38	3.765896	30.33843
EVX1	3.792125	3.728957
AC018553.1	3.881576	2.005592
FGL2	3.945182	229.7573
HAPLN1	3.956396	13.69715
ISM1	3.978062	3.237053
TLX3	3.983915	6.322412
PRDM13	4.021243	5.281042
LRP2	4.165894	64.82472
GREB1L	4.185683	67.49845
TFAP2A	4.232427	83.78614
ANGPTL7	4.342276	4.044429
AL359091.1	4.394286	4.160063
SOX14	4.403268	2.732075
GSX1	4.421251	4.187597
EVX2	4.481713	42.65978
HSPB3	4.531394	3.019723
DMBX1	4.587902	9.481603
SOST	4.780471	3.540895
ERG	4.795214	6.993186
EPYC	4.824933	25.47143
PAX7	5.045756	48.09391
ZPLD1	5.090629	2.913596
PRIMA1	5.115363	2.577295
RUNX3	5.236964	3.159005
CCDC140	5.363191	4.761266
TFAP2B	5.449415	131.3324
GBX2	5.538795	7.859802
TFAP2D	5.73966	3.456151
SKOR2	5.893214	18.17301
ATOH1	5.896612	3.774458
EVX1-AS	6.097905	4.178573
POU4F1	6.281595	21.6899
OLIG1	6.563678	5.06459
BARHL1	7.015813	18.65044

Supplementary Table 5. List of Antibodies used for immunohistochemistry

Antigen	Host Species	Source	Cat#	Dilution (WB)	Dilution (IHC)
P21	Mouse	Cell Signaling	2946	N/A	1:500
PAX6	Mouse	Santa Cruz	Sc-81649	N/A	1:500
CTIP2	Rabbit	Abcam	ab18465	N/A	1:500
KLOTHO	Rabbit	Bioss	bs-2925R	1:1000	1:500
TUJ1	Rabbit	Cell Signaling	D71G9	N/A	1:1000
TUJ1	Mouse	Sigma	T8578	N/A	1:1000
NEUN	Rabbit	Millipore	ABN78	N/A	1:500
TRA-1-60	Mouse	Cell Signaling	4746	N/A	1:500
SOX2	Mouse	Cell Signaling	L1D6A2	N/A	1:500
SOX2	Rabbit	Cell Signaling	D9B8N	N/A	1:500
NESTIN	Mouse	Cell Signaling	33475	N/A	1:500
GFAP	Rat	ThermoFisher	13-0300	N/A	1:500
ACTIN	Mouse	Bio-Rad	vma00048	1:5000	N/A

IHC: Immunohistochemistry, WB: Western Blot, N/A: Not Applicable

Supplementary Table 6. List of Primer Sequences used for RT-PCR (5′−3′ orientation).

Gene	Primer (Forward)	Primer (Reverse)
KL	AGGGTGCGTCCATCTGGGATA	GGGTTGTCGATGGTGATCCAG
p16	GTGGACCTGGCTGAGGAG	CTTTCAATCGGGGATGTCTG
IL-8	TTGGCAGCCTTCCTGATTTC	TCTTTAGCACTCCTTGGCAAAAC
IL-1α	GGTTGAGTTTAAGCCAATCCA	TGCTGACCTAGGCTTGATGA
IL-1β	CTCTCTCCTTTCAGGGCCAA	GAGAGGCCTGGCTCAACAAA
COL20A1	CCCCTCAAGTATCTGATCGTTTG	GAGACCAGGTACTCTGTGCG
SPARCL1	CCAACTGAAGGTACATTGGACAT	CTGTGAAGGAACTAACACCAGG
FGL2	AGATTGCTCTGACTACTACGCA	TGCCATGTTCTGGTGAAGTTG
GPC3	ATTGGCAAGTTATGTGCCCAT	TTCGGCTGGATAAGGTTTCTTC
TWIST1	GTCCGCAGTCTTACGAGGAG	GCTTGAGGGTCTGAATCTTGCT
MEIS1	GATATAGCCGTGTTCGCCAAA	CGGTGGCAGAAATTGTCACAT
GAPDH	AGCCACATCGCTCAGACAC	GCCCAATACGACCAAATCC

Supplementary blots

All blots derive from the same experiment and were processed in parallel

Fig. 1f

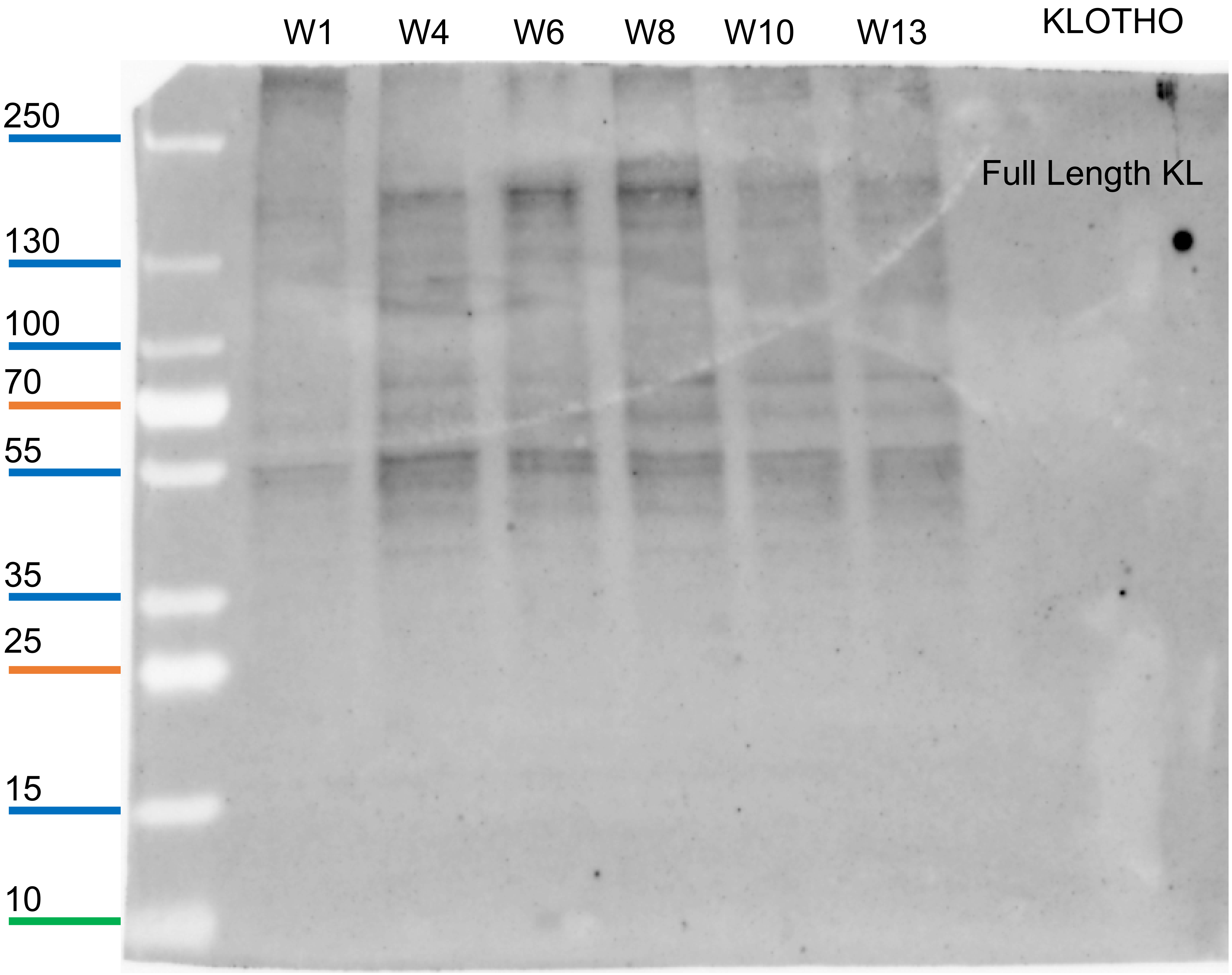
W1
W4
W6
W8
W10
W13
KLOTHO
Full Length KL
250
130
100
70
55
35
25
15
10

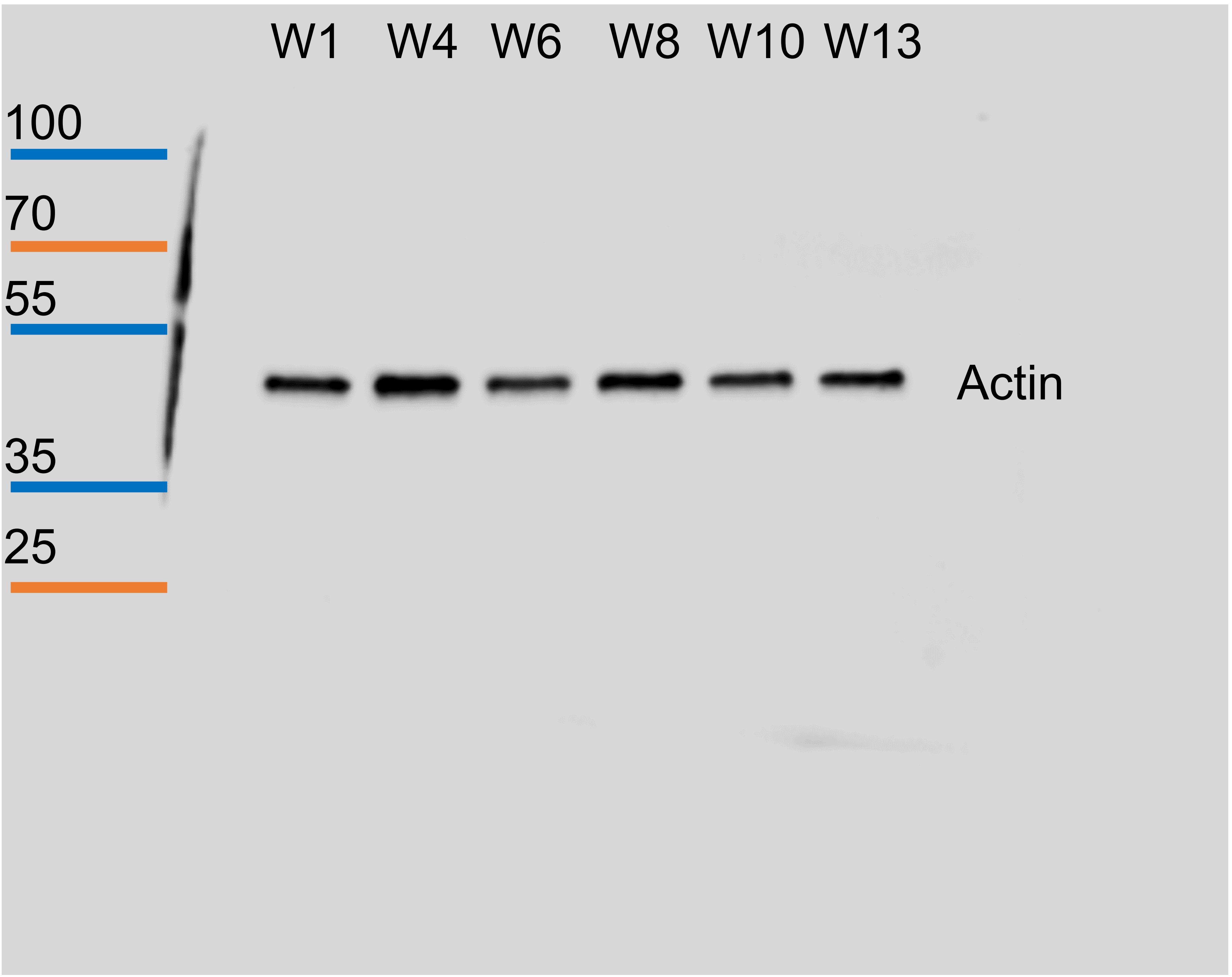
W1
W4
W6
W8
W10
W13
100
70
55
35
25
Actin

Fig. 1f

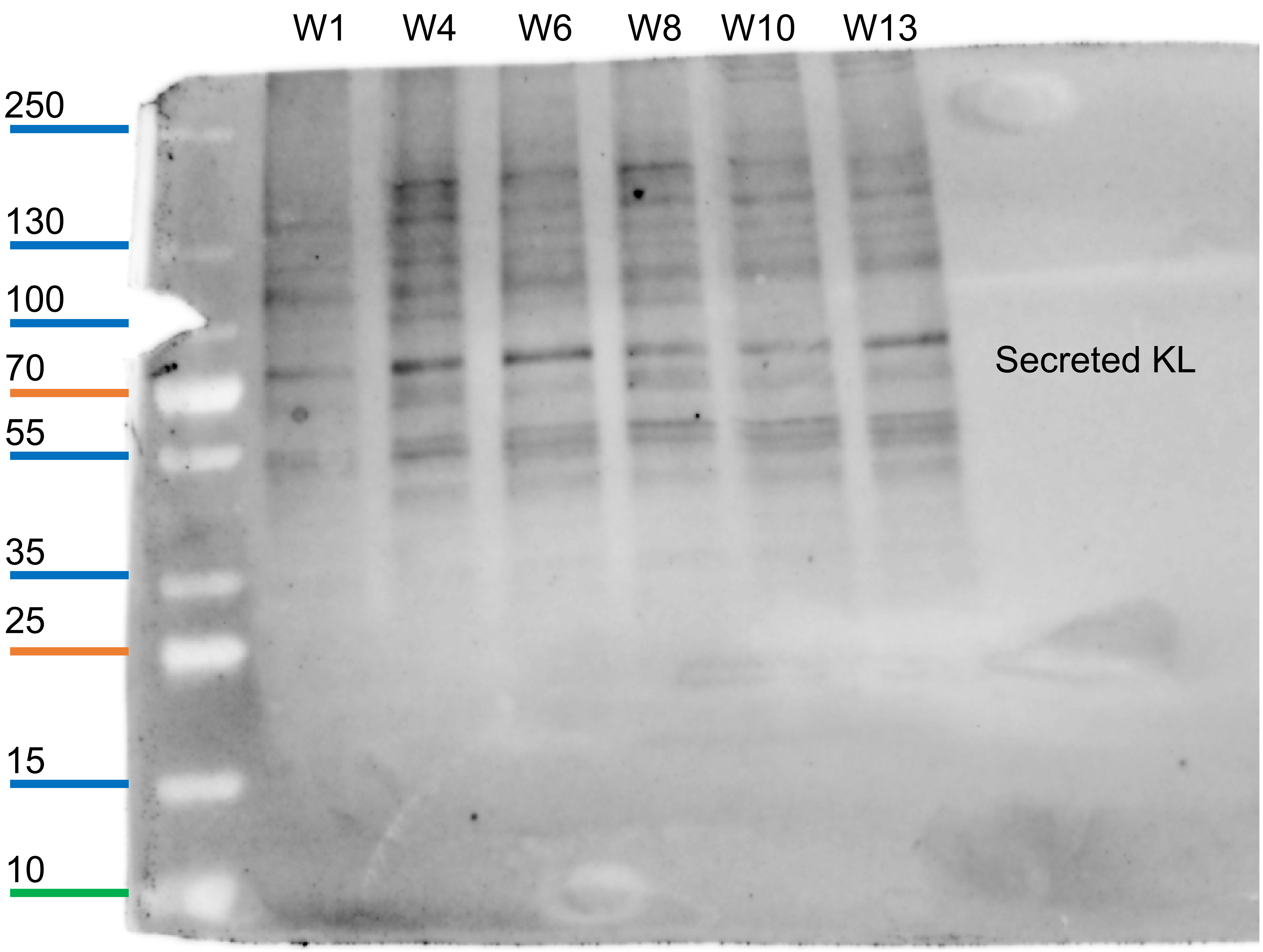

W1
W4
W6
W8
W10
W13
250
130
100
70
55
35
25
15
10
Secreted KL

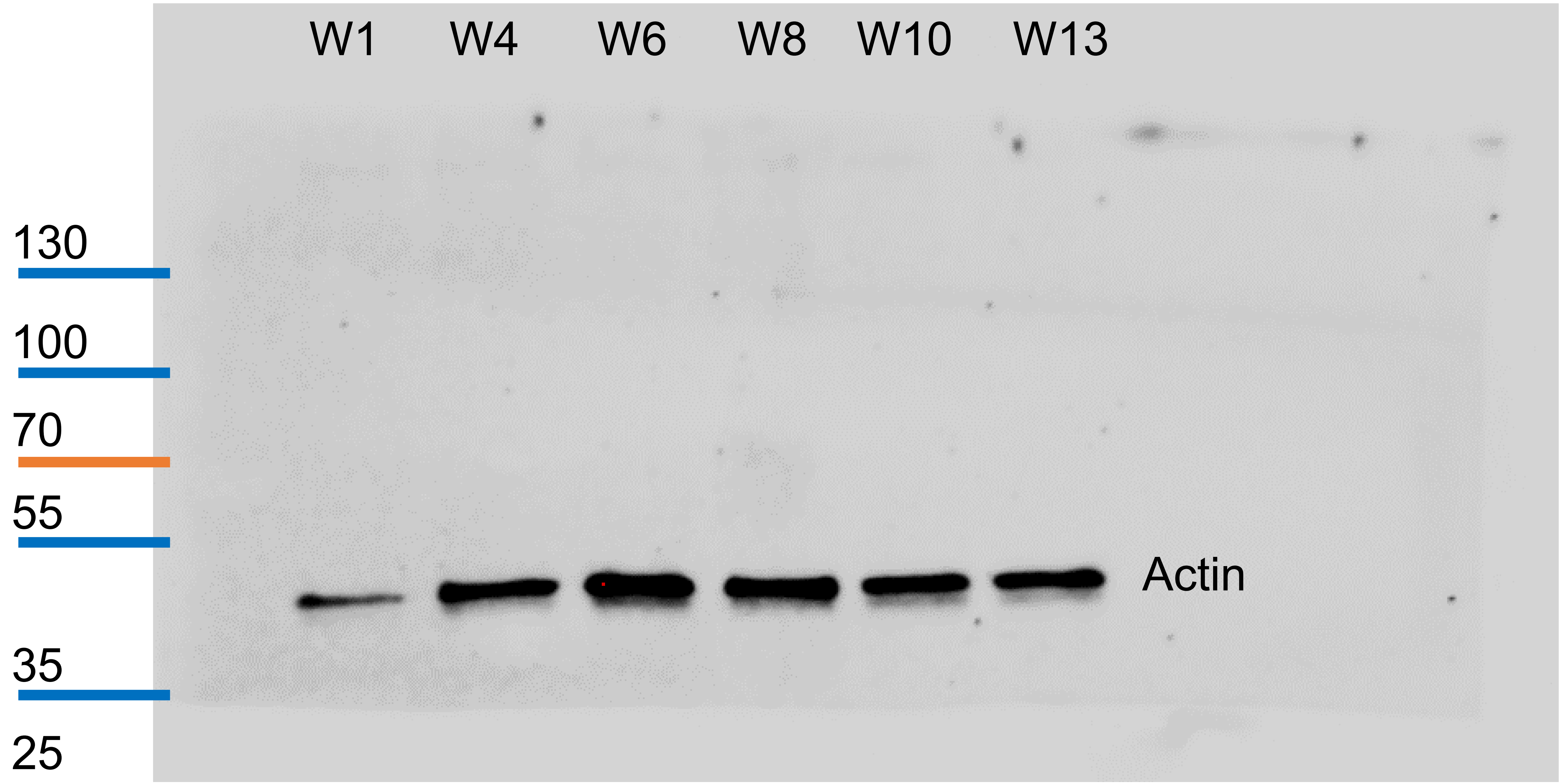

W1
W4
W6
W8
W10
W13
130
100
70
55
35
25
Actin

Supplementary Fig. 2b

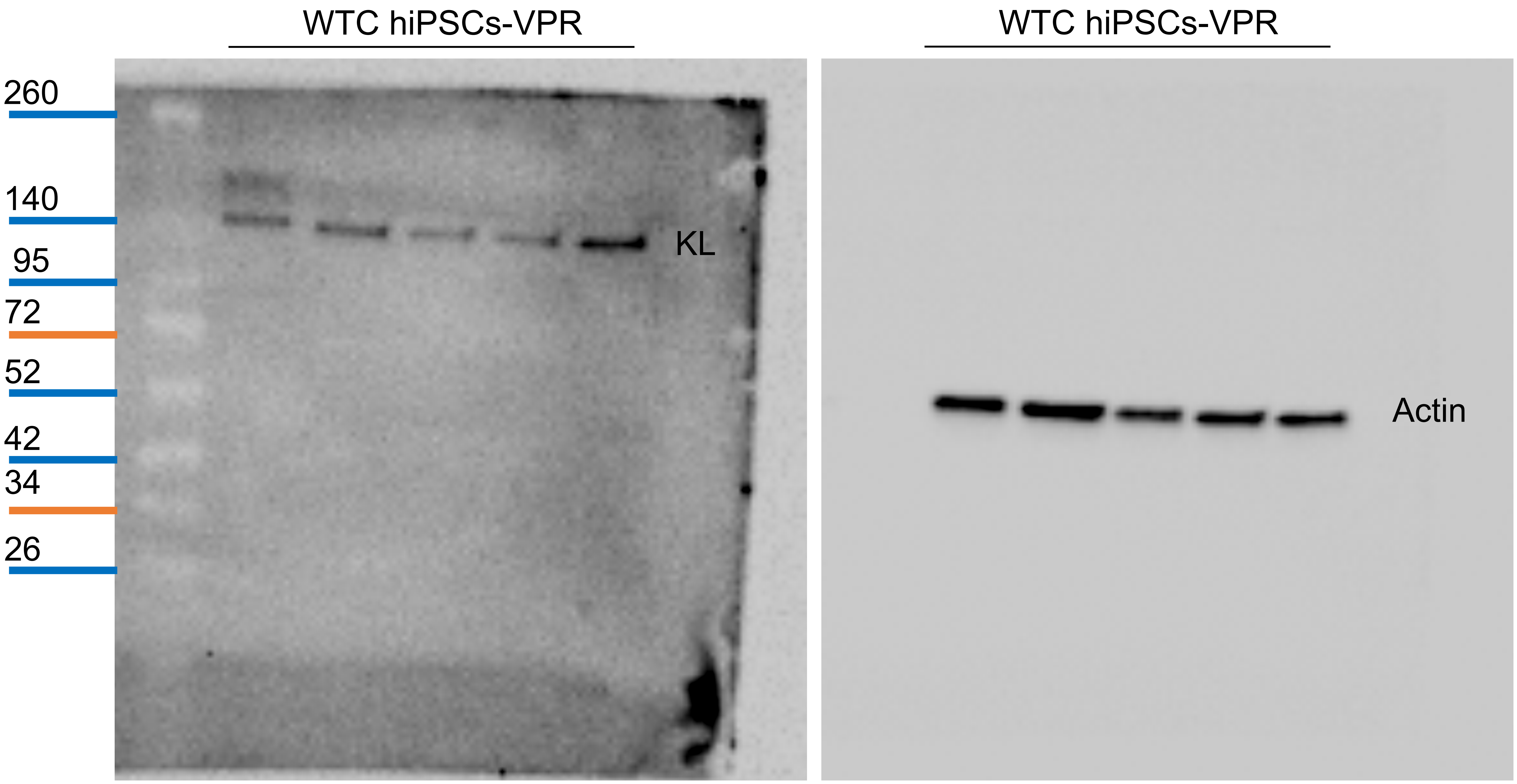

Supplementary Fig. 2b

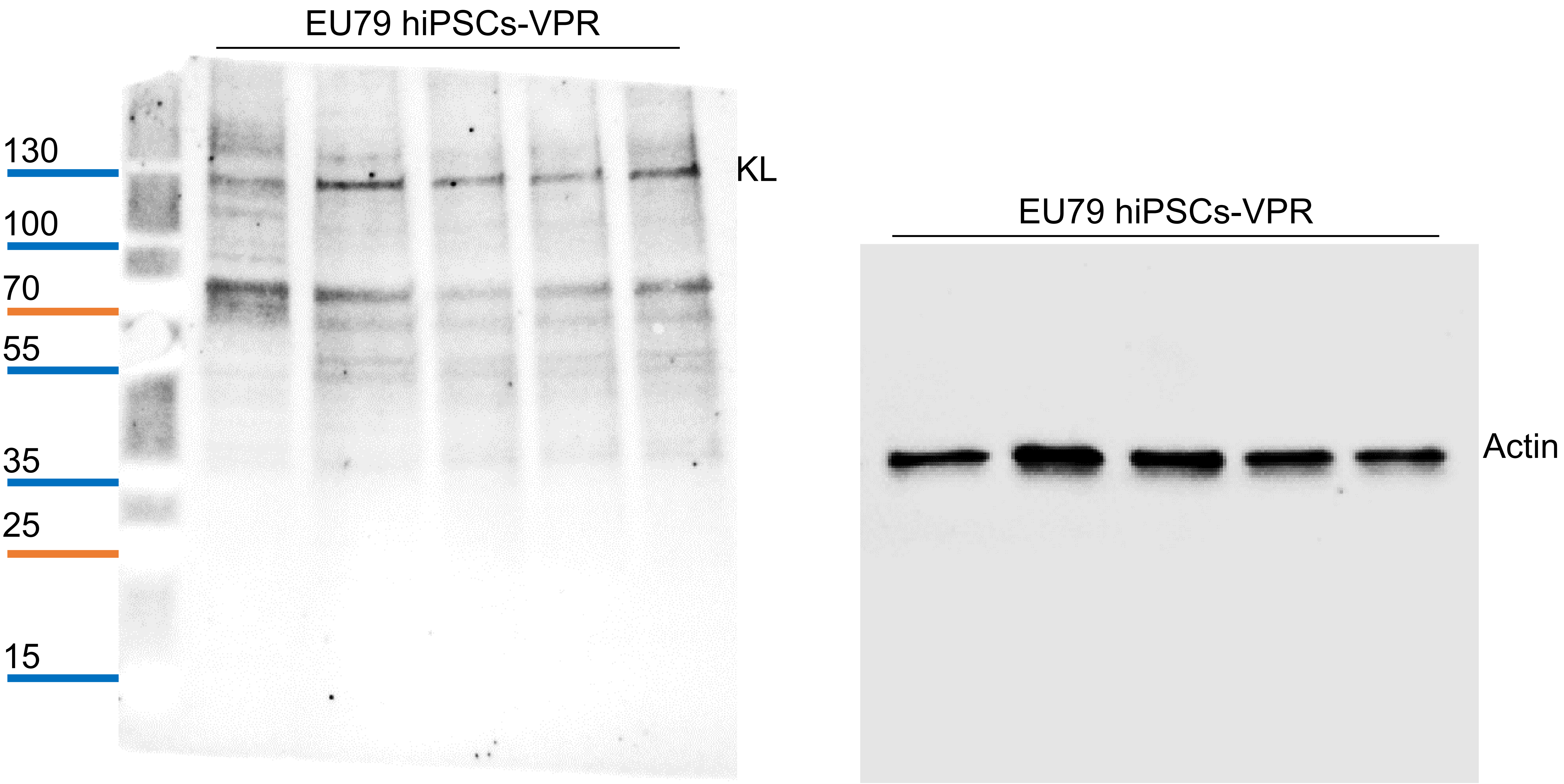

Supplementary Fig. 4b

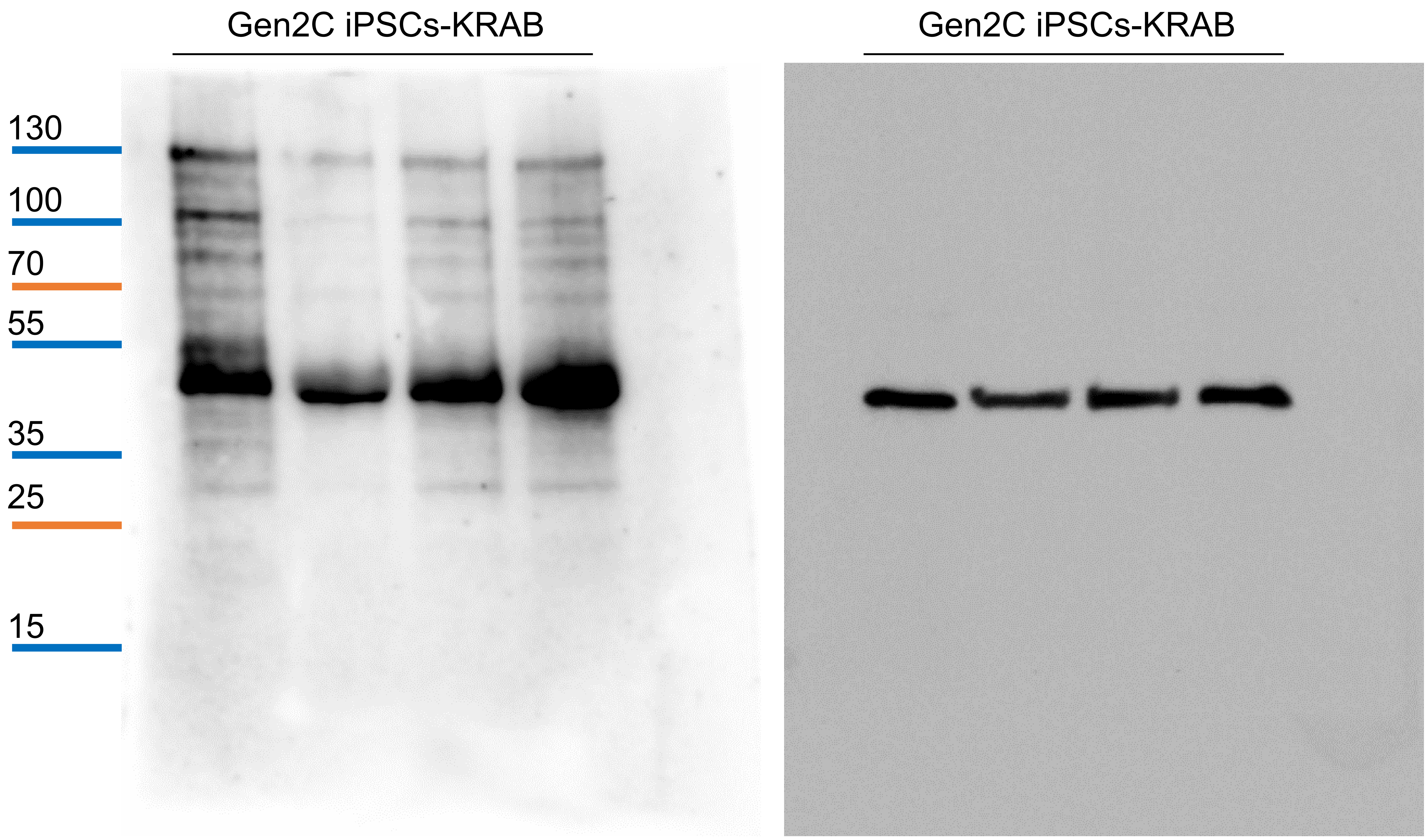

Supplement: Supplementary file 1 — Supplementary Information [file 41514_2021_70_MOESM1_ESM.pdf]
