# Supplementary material for: Klotho inhibits neuronal senescence in human brain organoids
Source: NPJ Aging Mech Dis. 2021 Aug 2;7:18. doi: 10.1038/s41514-021-00070-x (PMC8329278; doi:10.1038/s41514-021-00070-x)
Supplement: Supplementary file 4 — Reporting Summary [file 41514_2021_70_MOESM4_ESM.pdf]

nature research

Corresponding author(s): ernst wolvetang

Last updated by author(s): May 14, 2021

Reporting Summary

Nature Research wishes to improve the reproducibility of the work that we publish. This form provides structure for consistency and transparency in reporting. For further information on Nature Research policies, see our Editorial Policies and the Editorial Policy Checklist.

Statistics

For all statistical analyses, confirm that the following items are present in the figure legend, table legend, main text, or Methods section.

n/a	Confirmed	
☐	☒	The exact sample size (n) for each experimental group/condition, given as a discrete number and unit of measurement
☐	☒	A statement on whether measurements were taken from distinct samples or whether the same sample was measured repeatedly
☐	☒	The statistical test(s) used AND whether they are one- or two-sided<br>*Only common tests should be described solely by name; describe more complex techniques in the Methods section.*
☐	☒	A description of all covariates tested
☐	☒	A description of any assumptions or corrections, such as tests of normality and adjustment for multiple comparisons
☐	☒	A full description of the statistical parameters including central tendency (e.g. means) or other basic estimates (e.g. regression coefficient) AND variation (e.g. standard deviation) or associated estimates of uncertainty (e.g. confidence intervals)
☐	☒	For null hypothesis testing, the test statistic (e.g. F, t, r) with confidence intervals, effect sizes, degrees of freedom and P value noted<br>*Give P values as exact values whenever suitable.*
☒	☐	For Bayesian analysis, information on the choice of priors and Markov chain Monte Carlo settings
☒	☐	For hierarchical and complex designs, identification of the appropriate level for tests and full reporting of outcomes
☒	☐	Estimates of effect sizes (e.g. Cohen's d, Pearson's r), indicating how they were calculated

Our web collection on statistics for biologists contains articles on many of the points above.

Software and code

Policy information about availability of computer code

Data collection	NA
Data analysis	NA

For manuscripts utilizing custom algorithms or software that are central to the research but not yet described in published literature, software must be made available to editors and reviewers. We strongly encourage code deposition in a community repository (e.g. GitHub). See the Nature Research guidelines for submitting code & software for further information.

Data

Policy information about availability of data

All manuscripts must include a data availability statement. This statement should provide the following information, where applicable:

- Accession codes, unique identifiers, or web links for publicly available datasets
- A list of figures that have associated raw data
- A description of any restrictions on data availability

The data that support the findings of this study are available from the corresponding author upon reasonable request. Bulk RNA-sequence data from brain organoids that support the findings of this study have been deposited in the GEO-NCBI with the primary accession code GSE171719.

Field-specific reporting

Please select the one below that is the best fit for your research. If you are not sure, read the appropriate sections before making your selection.

☒ Life sciences ☐ Behavioural & social sciences ☐ Ecological, evolutionary & environmental sciences

For a reference copy of the document with all sections, see nature.com/documents/nr-reporting-summary-flat.pdf

Life sciences study design

All studies must disclose on these points even when the disclosure is negative.

Sample size	Minimal statistical significance was defined at $P < 0.05$.
Data exclusions	No data were excluded.
Replication	The minimum number of biological replicates is 3.
Randomization	Samples were randomly allocated into the groups.
Blinding	investigators were blinded for allocating samples and data analysis.

Reporting for specific materials, systems and methods

We require information from authors about some types of materials, experimental systems and methods used in many studies. Here, indicate whether each material, system or method listed is relevant to your study. If you are not sure if a list item applies to your research, read the appropriate section before selecting a response.

Materials & experimental systems

n/a	Involved in the study	
☐	☒	Antibodies
☐	☒	Eukaryotic cell lines
☒	☐	Palaeontology and archaeology
☒	☐	Animals and other organisms
☒	☐	Human research participants
☒	☐	Clinical data
☒	☐	Dual use research of concern

Methods

n/a	Involved in the study	
☒	☐	ChIP-seq
☒	☐	Flow cytometry
☒	☐	MRI-based neuroimaging

Antibodies

Antibodies used

P21 Mouse Cell Signaling 2946
PAX6 Mouse Santa Cruz Sc-81649
CTIP2 Rabbit Abcam ab18465
KLOTHO Rabbit Bioss bs-2925R
TUJ1 Rabbit Cell Signaling D71G9
TUJ1 Mouse Sigma T8578
NEUN Rabbit Millipore ABN78
TRA-1-60 Mouse Cell Signaling 4746
SOX2 Mouse Cell Signaling L1D6A2
SOX2 Rabbit Cell Signaling D9B8N
NESTIN Mouse Cell Signaling 33475
GFAP Rat ThermoFisher 13-0300
ACTIN Mouse Bio-Rad vma00048

Validation

Antibody specificity is confirmed by looking at cells that either do or do not express the target protein within the same tissue.

Eukaryotic cell lines

Policy information about cell lines

Cell line source(s)

Human embryonic stem cells H9 from (Wisconsin International Stem Cell Bank, WiCell Research Institute, WA09 cells), WTC iPSC (gift from Professor Bruce Conklin), EU79 iPSCs (published stem cell reports 2018). KRAB-GEN2C iPSCs lines (published Mandegar, M. A. et al. cell stem cell 2016), 293FT cells (Thermofisher Scientific, Cat. # R70007).

Authentication	Mycoplasma contamination was routinely checked in our cell lines of the laboratory
Mycoplasma contamination	We confirm all cell lines are mycoplasma negative.
Commonly misidentified lines<br>(See ICLAC register)	NA
